# Insights into the Ancient Adaptation to Intertidal Environments by Red Algae Based on a Genomic and Multiomics Investigation of *Neoporphyra haitanensis*

**DOI:** 10.1093/molbev/msab315

**Published:** 2021-11-03

**Authors:** Haimin Chen, Jeffrey Shih-Chieh Chu, Juanjuan Chen, Qijun Luo, Huan Wang, Rui Lu, Zhujun Zhu, Gaigai Yuan, Xinxin Yi, Youzhi Mao, Caiping Lu, Zekai Wang, Denghui Gu, Zhen Jin, Caixia Zhang, Ziyu Weng, Shuang Li, Xiaojun Yan, Rui Yang

**Affiliations:** 1 State Key Laboratory for Managing Biotic and Chemical Threats to the Quality and Safety of Agro-products, Ningbo University, Ningbo, China; 2 Marine Drugs and Biological Products Department, Ningbo Institute of Oceanography, Ningbo, China; 3 Wuhan Frasergen Bioinformatics Co. Ltd., Wuhan, China; 4 Collaborative Innovation Center for Zhejiang Marine High-efficiency and Healthy Aquaculture, Ningbo University, Ningbo, China; 5 Ningbo Customs Technology Center, Ningbo, China

**Keywords:** *Neoporphyra haitanensis*, genome, multiomics, intertidal adaption, desiccation

## Abstract

Colonization of land from marine environments was a major transition for biological life on Earth, and intertidal adaptation was a key evolutionary event in the transition from marine- to land-based lifestyles. Multicellular intertidal red algae exhibit the earliest, systematic, and successful adaptation to intertidal environments, with *Porphyra* sensu lato (Bangiales, Rhodophyta) being a typical example. Here, a chromosome-level 49.67 Mb genome for *Neoporphyra haitanensis* comprising 9,496 gene loci is described based on metagenome-Hi-C-assisted whole-genome assembly, which allowed the isolation of epiphytic bacterial genome sequences from a seaweed genome for the first time. The compact, function-rich *N. haitanensis* genome revealed that ancestral lineages of red algae share common horizontal gene transfer events and close relationships with epiphytic bacterial populations. Specifically, the ancestor of *N. haitanensis* obtained unique lipoxygenase family genes from bacteria for complex chemical defense, carbonic anhydrases for survival in shell-borne conchocelis lifestyle stages, and numerous genes involved in stress tolerance. Combined proteomic, transcriptomic, and metabolomic analyses revealed complex regulation of rapid responses to intertidal dehydration/rehydration cycling within *N. haitanensis*. These adaptations include rapid regulation of its photosynthetic system, a readily available capacity to utilize ribosomal stores, increased methylation activity to rapidly synthesize proteins, and a strong anti-oxidation system to dissipate excess redox energy upon exposure to air. These novel insights into the unique adaptations of red algae to intertidal lifestyles inform our understanding of adaptations to intertidal ecosystems and the unique evolutionary steps required for intertidal colonization by biological life.

## Introduction

The initial diversification of macroalgae appears to have occurred in the Mesoproterozoic (Bykova et al. 2020). Subsequent fluctuations of Earth’s climate and geologic evolution impacted the formation of intertidal zone habitats. Many organisms would have then been passively dragged from shallow waters to intertidal zones. Those that survived in these zones would be required to overcome many challenges including desiccation, daily and seasonally fluctuating temperatures, high levels of irradiance, and severe osmotic stress (Wang et al. 2020). Around this time, some red algae, which are considered among the earliest members of the Archaeplastida (Brawley et al. 2017), were also introduced to intertidal zones and encountered the harsh conditions associated with them. The surviving red algae would have thus successfully competed in this dynamic and stressful environment for over the next billion years, while acquiring various intertidal adaptive traits.

*Porphyra* sensu lato within the Bangiales order of Rhodophyta is a typical exemplar of the adaptive evolution of organisms that transitioned from marine to early intertidal zone habitats (Yang et al. 2020). Evidence supporting this comes from the Bangia-like fossil *Bangiomorpha pubescens* (dated to 1,198 ± 24 My), which was inferred to live in intertidal zone habitats during ancient eras (Butterfield 2000; Blouin et al. 2011). Several aspects of intertidal adaptation can be observed in modern *Porphyra*. First, *Porphyra* exhibit adaptations to intertidal recurrent desiccation/rehydration cycling. They have developed the ability to undergo nearly absolute dehydration during air drying (losing up to 95% of their water), but become metabolically active as soon as they are rehydrated from rising tides (Brawley et al. 2017). Second, *Porphyra* exhibit unique two-generation-altered life histories of gametophyte thalli and sporophyte conchocelis (Wang et al. 2020). The ancestors of Bangiales may have adopted a unique shell-borne conchocelis lifestyle to obtain a stable, aqueous environment to mediate the transition from a water-borne environment to intertidal zones. However, the demand for population diversity and expansion would have required them to enter the intertidal environment and initiate thallus generation. Therefore, thallus is a typical adaptive state for *Porphyra* under harsh intertidal conditions.

Desiccation tolerance (DT) of intertidal algae is similar to the desiccation resuscitation behavior of some plants. They all have the remarkable capacity to survive complete dehydration and come alive when water becomes available (Xiao et al. 2015). However, revival mechanisms differ considerably among organisms. Resurrection plants, including ferns, mosses, and a few angiosperm plants, can tolerate desiccation for days, months, or even years (Lüttge et al. 2011). Responses to desiccation have been intensively studied in resurrection plants, and observed strategies include increasing compatible solutes, strengthening antioxidant activity as a protective mechanism, using xanthophyll cycling to dissipate energy, and activating the abscisic acid (ABA) pathway (Lüttge et al. 2011). In contrast, intertidal macroalgae experience rapid desiccation and rehydration changes as a result of rises and falls in tides that occur once or twice a day. The responses of seaweeds to desiccation have mainly been characterized physiologically. Several studies have investigated the variation in photosynthetic parameters and antioxidant enzyme activities (Lipkin et al. 2009; Lüttge et al. 2011; Wang et al. 2020), although comprehensive analyses of their recurrent rapid revival mechanisms remain understudied.

The acquisition of DT by intertidal algae after encounters of drought stress may have evolved through genome modification and activation of some innate mechanisms that are ancestral to intertidal algae and may be conserved in land plants. Increasing numbers of high-quality Rhodophyte genome sequences have been generated in recent years (Matsuzaki et al. 2004; Bhattacharya et al. 2013; Collen et al. 2013; Lee et al. 2018; Cao et al. 2020) and these genomic resources will allow for a better understanding of the evolution of red algae. Consequently, comparative genomic analyses of these species will enable a systems approach for understanding the adaptations of the earliest organisms to intertidal zone habitats.

Here, we present a high-quality genome for *Neoporphyra haitanensis* (redefined from the synonym *Pyropia haitanensis* in May 2020) that is a typical upper intertidal species. Comparison of this genome against other red algae genomes was used to identify common evolutionary adaptations underlying their unique stress tolerance mechanisms. In addition, a comprehensive analysis of the changes in thalli during desiccation that were related to the revival mechanism was conducted using a multiomics approach. These data were used to propose an evolutionary scenario involving ancestral red algae that were exposed to ecological stress.

## Results and Discussion

### Metagenome-Hi-C-Assisted Genome Assembly of *N. haitanensis* and Removal of Assembly Microbial Contamination

**Fig. 1.**
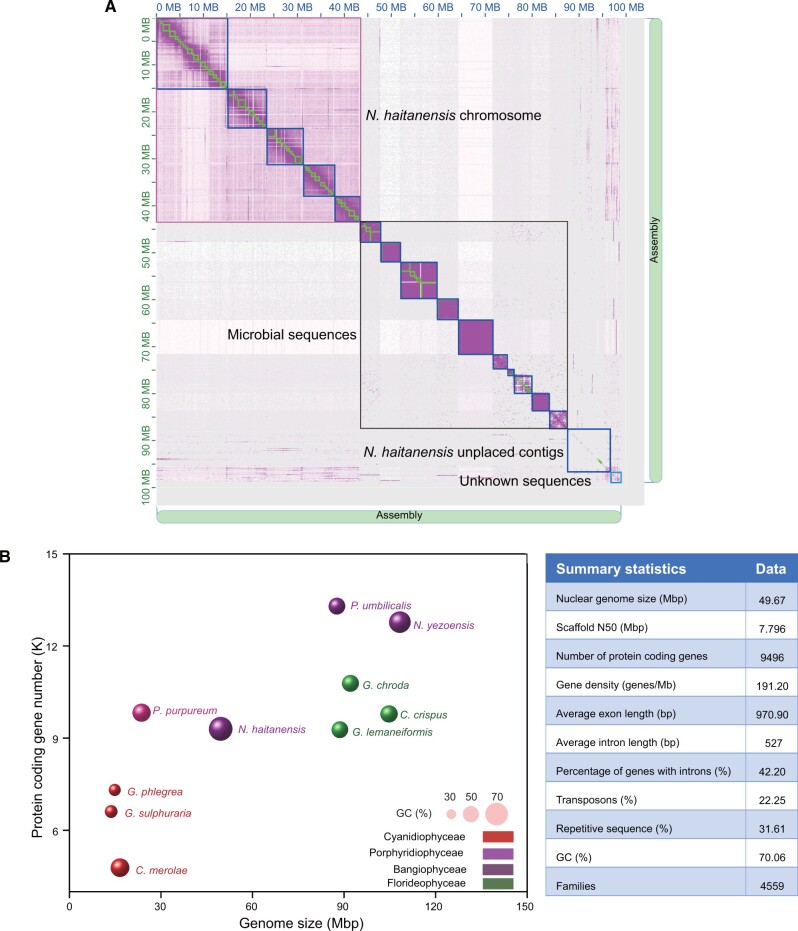
*Neoporphyra haitanensis* genome features. (*A*) Genome-wide Meta-Hi-C interaction links between the assembled contigs. Purple dots represent Meta-Hi-C interactions between two loci. (*B*) Summary statistics for the *N. haitanenssis* genome and comparison of genome size, GC content, and protein-coding gene numbers among red algae species.

Marine seaweeds often harbor bacterial populations tightly that cannot be completely removed by physical or antibiotic treatments. These close associations can cause significant obstacles when assembling seaweed genomes (Nakamura et al. 2013). The primary genome assembly of the *N. haitanensis* double haploid was generated using 19.32 Gb of PacBio long-read sequencing data, resulting in a 99.03 Mb genome comprising 15 scaffolds and 343 contigs (supplementary table 1, Supplementary Material online). Bacterial attachment was not found on the surface, inside of the cell wall or between the cell wall and plasma membrane by scanning and transmission electron microscopy (TEM) and staining observations (supplementary fig. 1, Supplementary Material online). However, previous research on 16S rRNA sequencing of antibiotic-treated thalli showed that bacteria were still present (Gu et al. 2020). Thus, to increase the accuracy of our genome assembly, we used metagenome-Hi-C (Meta-Hi-C) to separate the contigs of the target species from those of the attached microorganisms. Meta-Hi-C assembly cross-links DNA molecules that are in close physical proximity within intact cells (Lieberman-Aiden et al. 2009; Stewart et al. 2018). DNA interactions are stronger within the same DNA molecule than between DNA molecules, thereby allowing for the differentiation of host genome sequences from microbial genome sequences and resulting in species-level deconvolution (Burton et al. 2014). The Hi-C library comprised a total of 27 Gb of clean reads that were paired based on interaction information for different contigs to generate the Hi-C scaffolding data set. The contigs were separated into four groups (fig. 1*A*), with the first containing five scaffolds with strong intra-scaffold Hi-C interactions and minor inter-scaffold interactions. The five assembled scaffolds comprised the chromosomal region of *N. haitanensis*. The second group comprised ten scaffolds of strong intra-scaffold Hi-C interactions, and little or no interactions between scaffolds (fig. 1*A*). Comparison of these sequences against GenBank indicated that they resembled bacterial sequences. The third region was composed of short *N. haitanensis* unplaced contigs that were not able to cluster based on Hi-C data. The fourth group contained contigs that did not belong to *N. haitanensis* or to bacterial species (“unknown”) based on our BLASTn strategy (supplementary data 1, Supplementary Material online). The final *N. haitanensis* nuclear genome assembly, excluding contigs that were plastid, bacterial, and unknown, was 49.67 Mb, with contig N50 and scaffold N50 of 650 kb and 7.79 Mb, respectively (supplementary table 1, Supplementary Material online). Annotation and masking of repetitive elements resulted in 31.61% of the genome being masked (supplementary tables 2 and 3, Supplementary Material online). Combining ab initio prediction, homology-based prediction, and PacBio Iso-seq data, 9,496 protein-coding genes were predicted in the *N. haitanensis* genome (supplementary table 4, Supplementary Material online), of which 99.35% were supported by RNA sequencing (RNA-seq) data. Gene annotation completeness according to BUSCO analysis estimated that 85.8% of the core eukaryotic gene sets were found (supplementary table 5, Supplementary Material online). Comparing our results with *Porphyra umbilicalis* (Brawley et al. 2017) and *P.haitanensis* PH40 (Cao et al. 2020) genome assembly, we achieved a higher contiguity (supplementary table 6, Supplementary Material online). The smaller genome size and gene count also could be attributed to the use of Meta-Hi-C to separate non-*N. haitanensis* sequences.

Comparison of all available red algal genomic data, including genomes belonging to the Cyanidiophyceae, Bangiophyceae, and Florideophyceae groups, indicated that the *N. haitanensis* genome size was intermediate between those of Cyanidiophyceae and Florideophyceae, whereas the genome size and number of predicted genes were markedly lower for the Bangiophyceae genomes. The GC content in the *N. haitanensis* genome is 70.06%, which is higher than for other red algae (fig. 1*B*). Compact genes and high GC% are typical for this group, consistent with previous observations in other red algae and thus are possibly an ancestral trait (Brawley et al. 2017).

### Comparative Genomics Provides Evidence for Adaptive Intertidal Characteristics

**Fig. 2.**
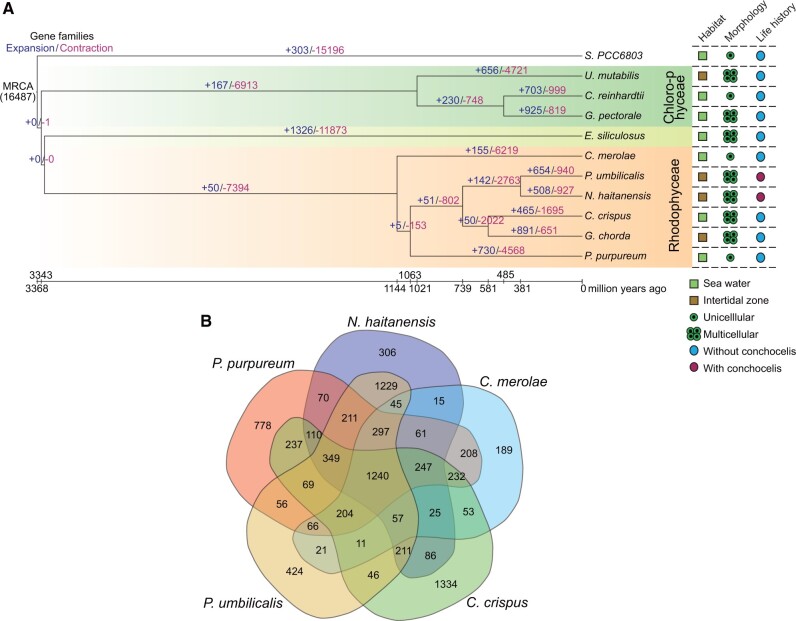
Comparative genomics analysis. (*A*) Comparative evolutionary histories of *Neoporphyra haitanensis* and other eukaryotic algae genomes. The number of gene families that were acquired (blue) or lost (purple) at each time point in the tree were estimated using the Dollo parsimony principle. The brown-shaded box in the phylogenetic tree represents algae that live in the middle or high intertidal zone and have desiccation adaptation features. (*B*) Venn diagram representation of shared and unique gene families in the *N. haitanensis* genome in the context of those common to red algae genomes, including those of *Porphyra umbilicalis*, *Porphyridium purpureum*, *Cyanidioschyzon merolae*, and *Chondrus crispus*.

Phylogenetic analysis of the estimated divergence of *Porphyra* (Bangiophyceae) and the clade comprising *Chondrus crispus* and *Gracilariopsis chorda* (Florideophyceae) from their common ancestor was dated to about 738.5 Ma (fig. 2*A*). Because all these taxa have intertidal characteristics, the adaptation of red algae to intertidal zones should have occurred before 738.5 Ma. To identify unique features of the *Porphyra* gene repertoire related to an intertidal lifestyle, the gene family content of *N. haitanensis* and *P. umbilicalis* was compared against that of other red algae. The common ancestor of these taxa is predicted to have gained 142 gene families that were associated with several categories (fig. 2*A* and supplementary data 2, Supplementary Material online), as follows: 1) gene families associated with unique shell-borne conchocelis life histories, including the presence of genes encoding carbonic anhydrase (CA), calcium-transporting ATPases, and calcium-dependent protein kinases; 2) genes associated with adaptation to intertidal environments, including redox-associated genes like catalase (CAT), glutathione S-transferase (GST), peroxiredoxin, and thioredoxin, in addition to stress-resistance-related genes, including those encoding germin-like protein, CBL-interacting protein kinase, disease resistance protein, subtilisin-like protease, and lipoxygenase (LOX); and 3) transporter genes, including ATP-binding cassette (ABC) transporters and copper-transporting ATPase. A total of 508 unique gene family gains were identified for *N. haitanensis* (fig. 2*A*), including a large number of expansions in antioxidant genes encoding glutamate synthase, GST, and haloperoxidases (supplementary data 3, Supplementary Material online).

We evaluated the genomes from several red algae with complete annotation information based on shared gene contents (fig. 2*B*). The *N. haitanensis* and *P. umbilicalis* genomes contained many common gene families that are specific for these two species, including 1,229 gene families (1,573 genes) (supplementary data 3, Supplementary Material online).

### Identification of Important Relationships between *N. haitanensis* and Associated Bacterial Populations Based on Meta-Hi-C Analysis

Meta-Hi-C assembly resulted in ten superscaffolds with contig N50 3.73 Mb and scaffold N50 4.44 Mb (supplementary table 7, Supplementary Material online). CheckM analysis for each of the ten superscaffolds showed eight superscaffolds with more than 98% completeness, whereas Superscaffold7 and Superscaffold8 showed 84.6% and 70.9% completeness, respectively. The estimated contamination in these superscaffolds were less than 1% in six superscaffolds. GTDB-Tk and PATRIC annotation also indicated these superscaffolds were of bacterial origin (supplementary data 4–6, Supplementary Material online). Whole-genome alignment showed that only some superscaffolds were highly homologous to genomes from known bacterial species (supplementary fig. 2*A*, Supplementary Material online). For example, Superscaffold1 aligned well to the genome of *Stappia* sp., Superscaffold4 was highly syntenic with the genome of *Maribacter* sp., and Superscaffold9 exhibited good synteny with the *Candidatus* Phaeomarinobacter ectocarpi genome (supplementary fig. 2*A*, Supplementary Material online). Other superscaffolds exhibited poor synteny with genomes of known bacterial species.

The sequence location mapping of the genes in the superscaffolds included a large number of unannotated sequences (supplementary fig. 2*B*, Supplementary Material online), potentially indicating the presence of a significant amount of genetic variation within the epiphytic bacterial genomes or the potential presence of novel bacterial genomic diversity. We also used the Distilled and Refined Annotation of Metabolism (DRAM) tool to see whether we could improve the annotation of these superscaffolds. Nearly all superscaffolds had full complements of prokaryotic electron transport chain (ETC) complexes, as well as genes in glycolysis pathway, pentose phosphate pathway, citrate cycle, glyoxylate cycle, and dicarboxylate hydroxybutyrate cycle (supplementary fig. 3, Supplementary Material online). Together, these results suggested that high-quality genomic assemblies based on Meta-Hi-C methods could inform the discovery of new bacterial species in samples from complex environments.

Increasing evidence has suggested that interactions between epiphytic bacteria and seaweeds play key roles in normal algal development (De Clerck et al. 2018). Here, a total of 32,229 genes were annotated among the bacterial scaffolds, but over 68.42% of the bacterial sequences did not exhibit good matches to genomes within public databases. Some cytokinin biosynthesis-associated genes of *Ca.* P. ectocarpi were identified on Superscaffold9, in addition to numerous genes related to the biosynthesis of biotin, pantothenate, coenzyme A, pyridoxine, and thiamine (supplementary data 7, Supplementary Material online). *Ca.* P. ectocarpi has been observed to associate with brown algal *Ectocarpus* cultures and to harbor numerous transporters that can help assimilate algal metabolites. Their genomes also encode several proteins likely to be involved in the synthesis of algal hormones like auxins and cytokinins, in addition to vitamins (Dittami et al. 2014). Eleven types of phytohormones have been identified in *N. haitanensis*, including ABA, auxin (IAA), and jasmonic acid (JA) (Song et al. 2017). Most of the genes involved in phytohormone synthesis, however, are not present in the *N. haitanensis* genome. Therefore, the synthesis mechanisms for these hormones remain unknown (Mikami et al. 2016). Nevertheless, only some of the genes involved in phytohormone synthesis pathways were identified in the ten bacterial superscaffolds (supplementary data 7, Supplementary Material online). We could not dismiss the fact that epiphytic microorganisms and seaweed hosts could cooperatively synthesize some phytohormones.

We found common sequences existed among epiphytic bacterial genomes of *N. haitanensis* and other *Porphyra* sequencing data. The PacBio sequencing data from *P. haitanensis* PH40 and *P. umbilicalis* were aligned to the bacterial superscaffolds. A clear bias in alignment was observed primarily on Superscaffolds1, 2, 7, 9, and 10 (supplementary fig. 4*A*, Supplementary Material online). A total of 1,090 gene sequences were shared by the bacterial superscaffolds and the *P. haitanensis* PH40 sequencing data. Among these, 664 gene sequences aligned more than 90% of their length, indicating the presence of numerous complete genes among these shared sequences. In addition, 248 sequences that belonged to annotated genes were also shared among the bacterial sequences from the *N. haitanensis* assembly and the two *Porphyra* genome sequencing data (supplementary data 8, Supplementary Material online). *N.haitanensis* and *P. haitanensis* PH40 were collected from different sites of coastal China, and both underwent aseptic treatments before genomic sequencing (Wang et al. 2020), whereas *P. umbilicalis* had a different taxonomy and a different collection location or time (Brawley et al. 2017); nevertheless, it still harbored similar bacterial sequences. One explanation for this observation is that these sequences are bacterial conserved sequences, or bacterial epiphytes with these sequences universally associated with seaweeds. It is likely, then, that some close interactions exist between them and seaweeds. Enrichment analysis identified significant over-representation (*P* < 0.05) of genes within the common 248 bacterial sequences that were associated with the KEGG pathways “membrane transport,” “signal transduction,” and “vitamins.” Furthermore, a large number of transporter genes were identified as enriched that are involved in the transport of Fe^3+^, inorganic phosphorus, polyamines, heavy metals, sodium ions, urea, amino acids, and other compounds. These transporter genes included those within the ABC, BCCT, MFS, and TRAP transporter families. Of the 248 bacterial sequences, 28 were ABC transporter genes. In fact, we observed that among the 1,090 bacterial gene sequences shared between *N. haitanensis* and *P. haitanensis* PH40, 10% were ABC transporter genes, 61 of which were from the same bacterial species (supplementary data 8, Supplementary Material online). The high proportion of ABC transporter genes among the bacterial sequences common to *Porphyra* and the large number of transporter genes observed in *N. haitanensis* (supplementary fig. 5, Supplementary Material online) and other red algal genomes, including those of *P. umbilicalis* and *G. sulphuraria* (Schönknecht et al. 2013; Brawley et al. 2017), suggests that transporters may provide a mechanism to rapidly accomplish metabolic adaptation to handle complex environmental stresses.

### Rapid Adaptation of *N. haitanensis* to the Dehydration/Rehydration Cycle

**Fig. 3.**
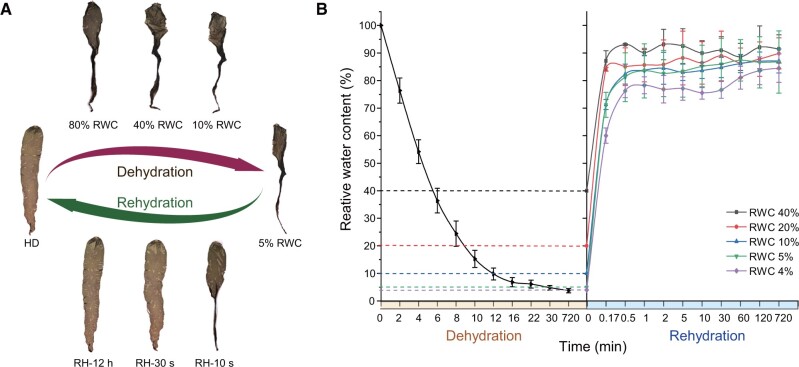
Phenotypic response of *Neoporphyra haitanensis* to intertidal desiccation and rehydration cycling. (*A*) Overview of desiccation and rehydration cycling of *N. haitanensis*. Phenotypic response to the desiccation and rehydration process of a single thallus. (*B*) Time course of RWC changes in thalli during a desiccation and rehydration cycle.

Once algae entered intertidal zone ecosystems, they were exposed to periods of desiccation/rehydration cycles. Here, we observed that thalli lost more than 95% of their water after desiccation for 30 min. Upon rewetting, thalli recovered 75% of their water content in 10 s, returning to almost normal in 2 min. Less water loss also led to faster recovery (fig. 3*B*). Intercellular space decreased during severe water loss, leading to cell shrinkage that was accompanied by extensive folding of cell walls. After rehydration for 12 h, cells had recovered from anhydrobiotic states. Across the entire recovery cycle, cell membranes and cell walls were closely connected, and thylakoids were always intact (supplementary fig. 6, Supplementary Material online). Even with drying up to 5% relative water content (RWC, 0.17 g ± 0.01 H_2_O/dry weight), almost no Evans blue or TUNEL positive staining within cells were observed, suggesting that *N. haitanensis* cells do not die during desiccation (supplementary fig. 7, Supplementary Material online).

### Multiomics Data Provide Evidence for Adaptation of *N. haitanensis* to Intertidal Environment Stresses

**Fig. 4.**
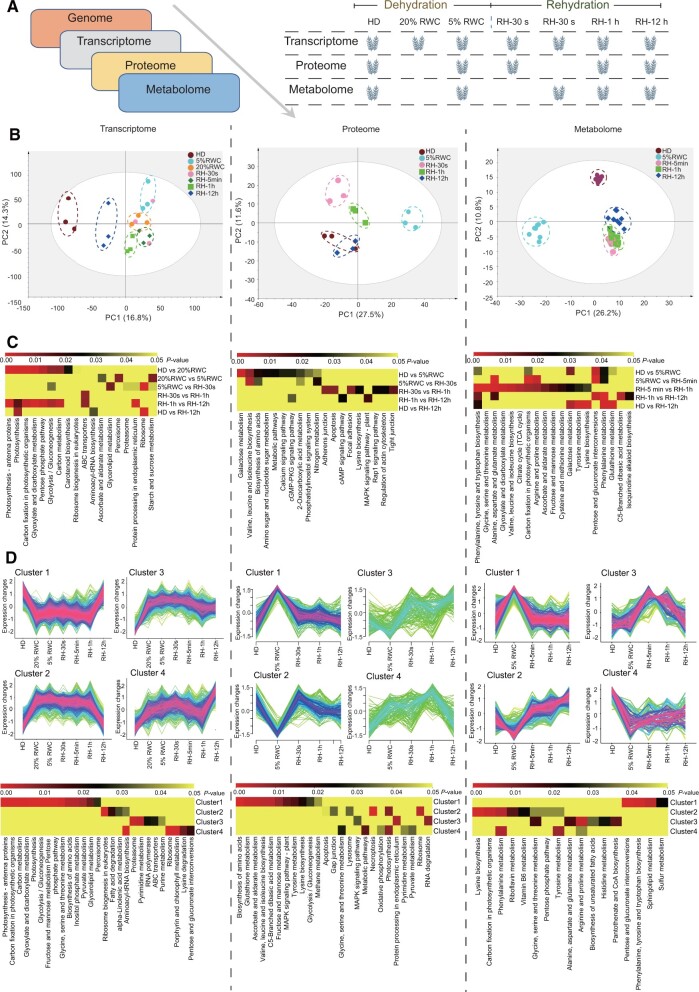
Multiomics analyses of the adaptations of *Neoporphyra haitanensis* to the desiccation and rehydration stress of intertidal zone habitats. (*A*) Overview of the multiomics study and the data sets that were used along with detailed descriptions of samples. (*B*) PCA of transcriptomic, proteomic, and metabolomic profiles of individual traits following shifts in dehydration and rehydration cycling. Circles around the same color points indicate biological replicates for each treatment. (*C*) Heatmaps of significantly enriched pathways for differentially variable genes, proteins, or metabolites across comparison groups. (*D*) Clusters of scaled transcript, protein, and metabolite profiles of *N. haitanensis* undergoing desiccation and rehydration cycling. The normalized DEGs, differentially expressed proteins, and differentially abundant metabolites were clustered into four groups. The corresponding heatmaps describe significantly enriched KEGG pathways for each cluster of the three omics data sets.

The transcriptional, proteomic, and metabolic responses of *N. haitanensis* to the dehydration/rehydration process was evaluated to understand their responses to intertidal environment stresses (fig. 4*A*). A total of 11,471 transcripts, 1,117 proteins, and 597 metabolites were identified, and principal components analysis (PCA) was performed to investigate the global responses at each functional level (fig. 4*B*). PCA revealed significant changes that occurred during water loss based on the three omics data sets, with great variation compared with the hydration (HD) (3.33 ± 0.10 g H_2_O/dry weight) group. Rehydration responses were much more rapid for the metabolites and proteins than for transcripts, and various levels of separation were observed between the 5% RWC (0.17 ± 0.01 g H_2_O/dry weight) and rehydration (RH)-5 min groups. However, transcripts separated very clearly after just 12 h of rehydration, suggesting that transcriptional recovery from dehydration was slowest among the other data sets. After 12 h of rehydration, proteins in RH-12 h clustered closely with the HD group, which indicated that proteins gradually recovered to a state close to that of HD group. Distances still existed between the RH-12 h and HD groups in transcriptomic PCAs, which indicated that some of the recovering reactions of the transcription process were occurring even after 12 h rehydration, such as “ribosome” and “aminoacyl-tRNA biosynthesis,” which meant that cells continued to prepare for the protein synthesis required for post-stress recovery, which is similar to some plants (Lüttge et al. 2011). The metabolites did not fully recover to the state before desiccation (fig. 4*C*). Various amino acid metabolism pathways in RH-12 h group were different from the HD group, such as tyrosine, lysine, glutathione, and phenylalanine metabolism, which also indicated that the intermediates of protein biosynthesis were still in an active state.

After drying, transcriptional changes were rapid and decreased from HD to 20% RWC (0.67 g H_2_O/dry weight) in 10 min, with the existence of about 2,000 differentially expressed genes (DEGs). However, when the water content decreased to 5%, the transcriptional state was maintained (with only 282 DEGs), indicating that the transcriptional changes primarily occurred in the early stages of water loss. Transcriptional levels did not respond quickly upon rehydration, with only 65 differential genes between the 5% RWC and RH-30 s (2.37 g H_2_O/dry weight) groups. When considering the difference between RH-1 h to RH-12 h, 1,855 DEGs were observed (supplementary fig. 8, Supplementary Material online). A transcript KEGG pathway enrichment heatmap indicated that dehydration favored gene transcripts involved in photosynthesis (i.e., those encoding photosynthesis-antenna proteins, photosynthesis, carbon fixation, and carotenoid biosynthesis genes) and glycometabolism (fig. 4*C*). Gene-co-expression analysis and clustering of the high-level DEGs into four major clusters was then conducted (fig. 4*D*). The pathways described above were all enriched in cluster 1 (*P* < 0.05) and significantly downregulated upon dehydration, indicating that photosynthesis shuts down quickly. The proteome exhibited a similar phenomenon, with proteins involved in photosynthesis being suppressed (cluster 2, *P* < 0.01). Dehydration from 20% RWC to 5% RWC led to the upregulation of transcripts involved in the “peroxisome,” “ascorbate and aldarate metabolism,” and “starch and sucrose metabolism” pathways indicating that antioxidant and osmoprotectant pathways began to change in the later stages of dehydration. Proteome differences recapitulated these same changes, wherein the “glutathione metabolism” and “ascorbate and aldarate metabolism” pathways were both upregulated (*P* < 0.01). Furthermore, the “ribosome biosynthesis,” “ribosome,” “RNA polymerase,” and “aminoacyl-tRNA biosynthesis” pathways were strongly upregulated during dehydration and in early rehydration (clusters 2–4, *P* < 0.01). Between RH-1 h and RH-12 h, the downregulated pathways of photosynthesis and carbon fixation recovered to normal states. Of course, it is possible that these pathways would recover in a shorter time.

The heatmap of metabolite KEGG enrichment revealed that separation of the HD from 5% RWC metabolite profiles was driven by several metabolites including those involved in “carbon fixation,” “amino acid biosynthesis,” “glutathione metabolism,” and “pentose and glucuronate interconversions.” Metabolite cluster 2 suggested that “carbon fixation” and pathways for the metabolism of several amino acids (e.g., Lys, Phe, or Tyr) were downregulated as RWC decreased, but increased upon rehydration (*P* < 0.05). In addition, differences between the 5% RWC and RH-5 min treatments, extending to the 1 h treatment, were driven by several glycometabolism, “tricarboxylic acid cycle,” and “amino acid intermediate” metabolites, indicating increased metabolic activity after the initiation of hydration (fig. 4*B* and *C*). Several amino acid biosynthesis genes were also upregulated during dehydration and in the early stage of rehydration (fig. 4*D*).

### Horizontal Gene Transfer May Be Involved in Intertidal Evolution

The evolution of red algal genomes has proceeded from complex to simple and back to gradual complexity again, with changes closely tied to environmental shifts (Bhattacharya et al. 2018). One of the major contributor is frequent horizontal gene transfer (HGT) to introduce gene gains and to reconstruct a rich gene function repertoire that compensated for gene loss events (Schönknecht et al. 2013; Lee et al. 2018). To examine possible HGT events in *N. haitanensis*, we aligned *N. haitanensis* gene sequences with refseq protein database and assessed using Alien Index (AI) (Gladyshev et al. 2008; Fan et al. 2020). We found 522 genes with AI score > 0 and among them, 489 genes were residing on the chromosomes. By categorizing genes with AI score > 10 and clustering with bacterial sequences on a phylogenetic tree, 267 genes can be regarded as strongly evidenced horizontally transferred candidate genes (HGT candidates) (supplementary data 9 and fig. 9, Supplementary Material online). To analyze these candidates further, we constructed a genome-wide dehydration and rehydration response profile between DEGs (*P* < 0.05) and HGT candidates. The DEGs and HGT-candidate genes did not reveal any obvious clustering in the genome because they were distributed across the five superscaffolds (supplementary fig. 10*A*, Supplementary Material online). However, the majority of these genes were predominantly located on scaffolds that were in a gene-rich region, suggesting that past HGT events were prevalent and not acquired by recent evolutionary events based on the assumption that sufficient time had allowed for dispersal of the genes throughout the genome. The scattered distribution of DEGs across the genome also reflected that the intertidal tolerance of *N. haitanensis* was not introduced by a restructuring event, but rather via retooling of existing genetic elements.

Gene enrichment analysis of the HGT genes further revealed that *N. haitanensis* might have acquired the ability to adapt to environments through a series of HGT events from bacteria (supplementary fig. 10*B* and data 3, Supplementary Material online). For example, 25 HGT-candidate genes were involved in oxidative stress responses, including Cu/Zn superoxide dismutase (SOD), GST, dioxygenase, and haloperoxidase. Many of them were unique to the *N. haitanensis* and *P. umbilicalis* genomes, with some being upregulated during dried and rehydration stages. In addition, Cu/Zn SOD and GST genes have been significantly expanded within the *Porphyra* genomes (supplementary data 3 and fig. 11*A* and B, Supplementary Material online). Further phylogenetic analysis revealed that the Cu/Zn SOD genes from species of *Porphyra* and other red algae clustered together and with that from the marine cyanobacterium *Acaryochloris marina* MBIC 11017. The Awaji stain of *A. marina* is an epiphyte of red algae (Chan et al. 2007), suggesting that they might have evolved from a common marine bacterial ancestral donor (supplementary fig. 11*C*, Supplementary Material online).

CAs are important enzymes that enable Bangiaceae seaweeds to concentrate carbon and maintain inorganic carbon assimilation capacity (Wang et al. 2020). Seventeen CA genes were identified in the *N. haitanensis* genome, eight of which were apparently derived from HGT. Four α-CA genes were specific to *N. haitanensis* and *P. umbilicalis*, two of which were expanded in the ancestor of the two species (supplementary data 3, Supplementary Material online). A comparison of CA expansion in red, brown, and green algae indicated that α-CA significantly expanded in *Porphyra* (supplementary fig. 12*A*, Supplementary Material online), consistent with the unusual characteristics of shell-borne conchoceli life histories. Phylogenetic analysis was then conducted for the α-CA genes (supplementary fig. 12*B*, Supplementary Material online), wherein those from green algae, Cyanobacteria, and other bacterial groups formed an independent clade that differed from the α-CAs from red algae. α-CAs presumed to be of an HGT origin clustered with other α-CAs from two *Porphyra* species in one large clade, suggesting they were derived from a common ancestor, and limited to the Bangiaceae family. Synteny analysis was then performed using CA genes of *N. haitanensis*, *P. umbilicalis*, and *P. purpureum* (supplementary fig. 12*C*, Supplementary Material online). The CA genes were distributed across the five *N. haitanensis* chromosomes. Thirteen of the CAs comprised conserved orthologous groups with their counterparts in the *P. umbilicalis* genome, with six of these being putatively derived from HGT. However, only two HGT-derived CA genes (*α-CA4-1* and *β-CA7*) formed conserved orthologous groups in both *P. purpureum* and *P. umbilicalis* genomes, suggesting that they were obtained from the ancestral red alga by HGT.

Extensive gene transfer appears to have been key to the genomic evolution of the metabolically versatile intertidal red algae. Red algae contain unusually diverse oxylipins, but it has remained a mystery regarding how such complexity of oxylipin synthesis pathways arises given their limited gene inventory (Andreou et al. 2009). For example, only two genes encoding LOXs have been identified in *Chondrus* (Collen et al. 2013). Here, evidence was observed that *N. haitanensis* obtained a whole set of LOX gene family genes, with six LOX genes arising from HGT, and four of these were specific to *N. haitanensis* and *P. umbilicalis* (*Phlox1*, *2*, *3*, and *5*, supplementary data 3, Supplementary Material online). The LOX genes within the *N. haitanensis* genome exhibited significant expansion in addition to those of other intertidal red algae, including *P. umbilicalis* and *G. chorda* (supplementary fig. 13*A*, Supplementary Material online). Phylogenetic analyses of LOX genes revealed two independent clusters. *Phlox1* and *2* formed an independent clade with the LOX genes from other red algae and exhibited high homology to those from the two marine bacteria, including *Shewanella violaea*, suggesting that they might actually be derived from marine bacterial donors (supplementary fig. 13*B*, Supplementary Material online). Syntenic analysis indicated that four *Phlox* genes exhibited conserved orthologous groups with their counterparts in the *P. umbilicalis* genome. *Phlox5* and *Phlox3*, in addition to *Phlox2* and *Phlox1*, corresponded to one gene in each of the two contigs of the *P. umbilicalis* genome, indicating that the two large fragments that evolved from the ancestor underwent duplication events in the *N. haitanensis* genome. However, conserved orthologous groups were not observed in the *P. purpureum* genome, indicating that all of the LOX genes in *Porphyra* were obtained after divergence from the mesophilic unicellular red alga *P. purpureum* (supplementary fig. 13*C*, Supplementary Material online). Many *Phlox* genes were upregulated in the dehydration and rehydration conditions (supplementary fig. 13*D*, Supplementary Material online). In addition, free polyunsaturated fatty acid (PUFA) and PUFA-containing membrane lipid contents increased upon dehydration, indicating that the oxylipin pathway is likely to be activated under these conditions (supplementary fig. 14, Supplementary Material online). We previously observed that some LOX proteins in *N. haitanensis* are multifunctional enzymes. For example, PhLox1 possesses unusually high hydroperoxidelyase, LOX, and allene oxide synthase catalytic activities based on one catalytic domain of the protein (Chen et al. 2015). Consequently, these results suggest that *N. haitanensis* obtained the LOX gene family from marine bacteria by HGT, the genes underwent massive expansion, and were later modified to achieve multifunctional ability, potentially explaining the observed diversity of red algal oxylipins.

### Short-Term Shutdown of Photosynthesis to Avoid Photo-Damage

**Fig. 5.**
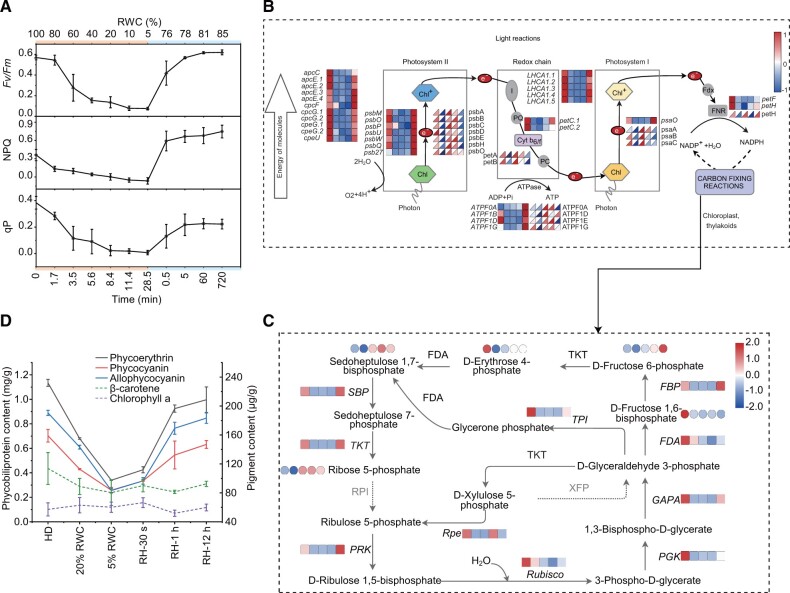
Strategies of *Neoporphyra haitanensis* photosynthetic systems in response to intertidal stress. (*A*) Photochemical efficiency of *N. haitanensis* during desiccation and rehydration cycling. (*B*) Transcriptome (squares) and proteome (triangles) expression profiles of the *N. haitanensis* photosynthetic system under dehydration and rehydration conditions are shown near the pathway as heatmaps. (*C*) Overview of the Calvin cycle response to osmotic stress. The data show transcriptome and proteome profiles from the HD (hydration), 5% RWC, RH (rehydration)-30 s, RH-1 h, and RH-12 h groups (from left to right). The square heatmaps show gene expression levels (normalized FPKM) (*n* = 3), the triangle heatmaps show the normalized peak areas of proteins (*n* = 8), and the circular heatmaps show the normalized peak area of metabolites (*n* = 8). Gray letters represent genes that have not been detected. (*D*) Changes in photosynthetic pigment content across dehydration and rehydration conditions (*n* = 3).

Red algae in intertidal zones experience periodic dryness every day. Their simple genomes have forced them to adopt passive, simple, and rapid modes of adaptation during desiccation. *N.haitanensis* shut down their photosynthetic activity through quenching *Fv*/*Fm* activities within minutes of transfer from fresh to desiccation conditions (fig. 5*A*). After rewetting, photosynthetic function was quickly regained within 30 s, with the *Fv/Fm* ratio recovering to 74% of that of fresh thalli and complete recovery within 5 min. The expression of numerous genes and proteins involved in photosynthesis were repressed during dehydration including nuclear-encoded components of the Lhca antenna complexes, PSI and PSII proteins, and F-type-ATPases (fig. 5*B*). However, protein expression was rapidly regenerated within 30 s of rehydration, whereas transcriptional levels continued to be inhibited until after 12 h of rehydration. *N.haitanensis* and *Neopyropia yezoensis* exhibit similar photo-inhibition phenomena under dehydration conditions (Wang et al. 2018). Huang et al. (2021) recently found that photosynthesis-antenna proteins, such as Lhca 1, were upregulated when *N. haitanensis* was under dehydration, and inferred that this is the strategy adopted by *N. haitanensis* to prevent or lower light-stress-induced damage. This finding is not consistent with our results and the speculation that *N. haitanensis* would shut down the photosystem to reduce the photo-damage.

In association with the shutdown of photosynthesis, the carbon fixation reactions of photosynthesis were also strongly repressed during dehydration. The *RuBP*, *Pgk*, and *Gapa* genes in the carboxylation and reduction stages of the Calvin cycle were downregulated, whereas most genes involved in the regeneration of ribulose 1,5-bisphosphate were also downregulated. Decreased abundances of detected metabolic intermediates confirmed this observation for the Calvin cycle (fig. 5*C*). Huang et al. (2021) also observed that carbon fixation pathways were significantly enriched and downregulated when RWC was decreased to 15%, and proposed that *N. haitanensis* responded to drought stress by reducing energy metabolism. We discovered that these changes almost completely reversed during the later period of rehydration, consistent with changes in *Fv/Fm* over the same period.

Another inevitable problem of intertidal life is photo-protection. *N.haitanensis* did not exhibit evident effects of photo-damage under dehydration conditions (supplementary figs. 6 and 7, Supplementary Material online). A nonphotochemical quenching (NPQ) mechanism implemented by the xanthophyll cycle is widely found in green algae and land plants that can protect the photosynthetic apparatus from photo-damage (Lüttge et al. 2011). Red algae are however generally not thought to have a xanthophyll cycle. For example, *Cyanidioschyzon merolae* lacks various mechanisms for dissipating excessive light energy (Matsuzaki et al. 2004). *Porphyra* exhibits similar deficiencies. When *N. haitanensis* was dehydrated, NPQ decreased, similar to the *Fv/Fm* trends (fig. 5*A*). Although several carotenoid derivatives were detected in *N. haitanensis*, including primarily zeaxanthin, β-carotene, and lutein (supplementary fig. 15, Supplementary Material online), most exhibited decreased concentrations. Violaxanthin, which participates in the xanthophyll cycle, was not detected, and interconversions of xanthophylls were not evident. Moreover, *N. haitanensis*, *P. umbilicalis*, and *N. yezoensis* all were observed to lack violaxanthin (Yang et al. 2014; Koizumi et al. 2018). Analysis of the *N. haitanensis* genome (along with other red algal transcriptomic analyses) also indicated that red algae lost the de-epoxidase gene (*Vde*); this loss was speculated to have occurred in red algae ancestors (Wang et al. 2018). Furthermore, evidence was not observed in the genome for some members of the PSII complex that are associated with NPQ, including Lhc SR, PsbS, Lhcx, and Lhcb4 (Lüttge et al. 2011), suggesting that *N. haitanensis* lacks a xanthophyll cycle. In contrast, we observed that the periodic photo-protection in *N. haitanensis* seems to rely on reducing all the outer light harvesting antenna pigments to avoid photo-damage caused by excessive photon absorption. During dehydration, significant decreases in the content of light-harvesting phycobiliproteins (phycoerythrin [PE], phycocyanin [PC], and allophycocyanin [APC]) were observed. In addition, the concentrations of the accessory pigment β-carotene were also diminished (fig. 5*D*). The core pigment chlorophyll a was retained throughout the hydration/dehydration process, which would help to maintain rapid recovery of photosynthesis upon rehydration. All content recovered 1 h after rewetting. Indeed, it is astonishing that photosynthetic system proteins can be decomposed and resynthesized in such rapid cycles. This is similar to the performance of poikilochlorophyllous (PDT) in plants, although PDT will dismantle their thylakoid membranes during dehydration and recovers slowly upon rewetting due to the time required for protein resynthesis (Lüttge et al. 2011). In contrast, the thylakoid membranes of dried *N. haitanensis* cells were intact based on TEM observation (supplementary fig. 6, Supplementary Material online). The mechanism underlying the rapid reconfiguration of the photosynthetic mechanism requires further study. However, this mechanism may be a deficiency of *Porphyra*. For example, because the photosynthetic system cannot properly initiate during low tide, they are unable to adapt to excessively long periods of desiccation, thereby preventing them from colonizing land habitats.

### S-adenosyl-l-Methionine-Dependent Methyltransferase Coordinates with Antioxidant System to Promote Intertidal Adaptation

**Fig. 6.**
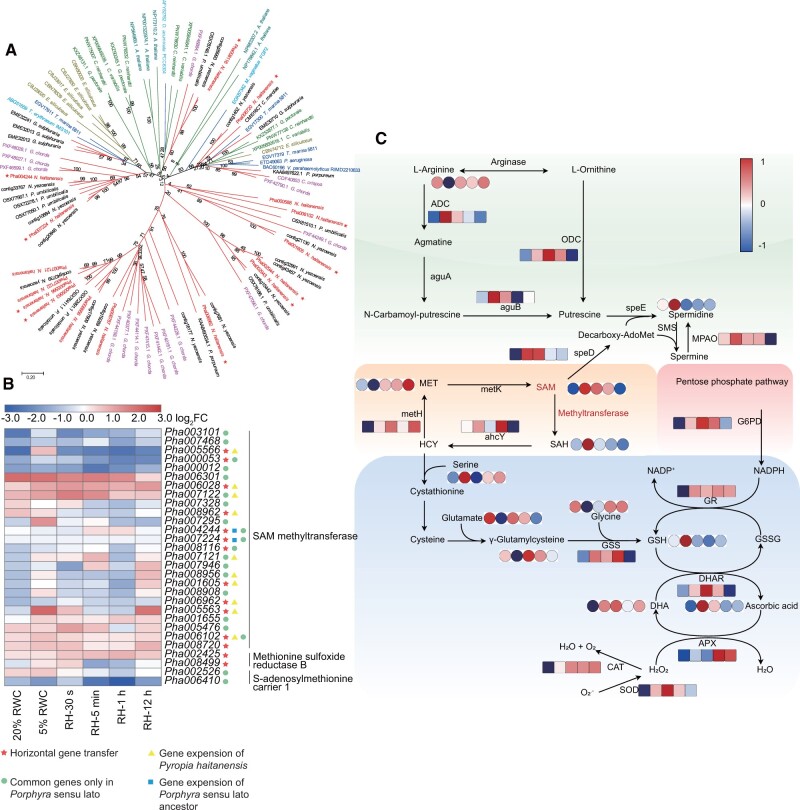
*Neoporphyra haitanensis* adapts to intertidal environments by enhancing SAM-dependent methyltransfer activity and synergistic activities with antioxidant systems. (*A*) Phylogenetic tree of SAM-dependent methyltransferase homologs from different species. Phylogenetic trees were constructed using neighbor-joining methods in MEGA7 and node support was evaluated with bootstrap tests (1,000 replicates). Red branches represent red algal genes, green branches represent plant genes (including genes from green algae and *Arabidopsis thaliana*), blue branches represent bacterial genes, and brown branches represent genes from *Ectocarpus siliculosus*. Homologs from the *N. haitanensis* genome are highlighted in red text, and members thought to derive from HGT are marked with a red star. (*B*) Heatmap showing differential expression of SAM-dependent methyltransferase genes and methyltransfer-related genes in *N. haitanensis* under desiccation and rehydration stress conditions. Values are fold-change (log2-ratio) values of transcript levels under dehydration and rehydration conditions relative to fresh conditions. (*C*) Summary of the SAM-polyamine-GSH-ascorbic acid metabolic network during desiccation and rehydration response by *N. haitanensis*. The network was reconstructed based on KEGG pathways. The squares in the heatmap show gene expression levels (normalized FPKM) (*n* = 3), and the circles in the heatmap show normalized average values of peak area for metabolites (*n* = 8) under hydration (HD), 5% RWC, RH (rehydration)-5 min, RH-1 h, and RH-12 h conditions (from left to right).

S-adenosyl-L-methionine (SAM)-dependent methylation plays several crucial roles in ribosomal stability (Wu et al. 2020); the biosynthesis of nucleic acids, proteins, and secondary metabolites (Luo et al. 2019); and in epigenetic regulatory processes (Sun et al. 2021). Numerous SAM-methyltransferase (SAM-MTase) genes were identified in the unique genes shared by *N. haitanensis* and *P. umbilicalis* and were also greatly expanded in the *N. haitanensis* genome. An additional 11 SAM-MTase genes were suspected to have been derived from HGT (supplementary data 3, Supplementary Material online). Phylogenetic analysis indicated that the SAM-MTase genes of *N. haitanensis* clustered with those of other *Porphyra* and were mainly distributed in two relatively independent clusters. The two clusters also comprised genes from the intertidal red alga *G. chorda*, suggesting that the transmethylation function was a specific adaptation of intertidal algae (fig. 6*A*). Many of these genes were upregulated during either early or late dehydration stages and during rehydration (fig. 6*B* and supplementary data 3, Supplementary Material online). Importantly, the unusually high SAM content increased by 6.07-fold in the 5% RWC group compared with their expression in fresh thalli (*P* < 0.01), whereas S-adenosylhomocysteine (SAH) content increased by 15.95-fold during dehydration (fig. 6*C*, *P* < 0.01). Demethylated SAH can be hydrolyzed by S-adenosyl-homocysteine hydrolase (ahcY) to generate homocysteine (HCY) that can then be remethylated to methionine (MET) by methionine synthase (metH) (Tehlivets et al. 2013). The *ahcY* and *metH* genes were upregulated under dehydration and rehydration conditions, indicating that SAH could be continuously recycled back to MET. Typically, 90% of SAM is involved in various methylation activities, whereas the remaining 10% is used for synthesizing polyamines (Moffatt and Weretilnyk 2001). Genes encoding enzymes involved in polyamine spermidine (SPD) synthesis exhibited significant upregulation (*P* < 0.01) that promoted the formation of SPD. Indeed, SPD exhibited significantly increased levels during dehydration (5.66-fold increase, *P* < 0.01). Importantly, we observed a particularly interesting SAM process coupling the glutathione (GSH)–ascorbic acid (AA) redox cycle to influence the antioxidant enzyme system in *N. haitanensis*. HCY accumulation can cause cell oxidative stress and is consequently recycled back to MET, but can also be converted to cysteine and then to GSH (Pizzorno 2014). Significantly increased GSH levels were observed in *N. haitanensis* during dehydration, with 45.13-fold higher levels than in fresh samples (*P* < 0.05). In addition, the entire GSH-AA-NADPH redox response system was highly activated during dehydration (*P* < 0.01), with AA exhibiting 433.59-fold higher levels than in the HD group (*P* < 0.01). Transient or sustained changes in all or some components of this system were all observed. Therefore, by cleverly combining the SAM-methyltransfer cycle, polyamine synthesis, and the GSH-AA-NADPH antioxidant system, a highly reducing intracellular context is created within cells that can help protect seaweeds from oxidative damage when exposed to falling tide environments.

### Rapid Biosynthesis and Repair of Macromolecules Is an Important Mechanism of Intertidal Adaptation

*N.* *haitanensis* responds to rehydration amazingly rapidly. The three different omics data sets analyzed here suggested that protein and metabolite levels rapidly responded to rehydration, but it is unclear how this is achieved. The basis for this rapid response is certainly due to adequate advance preparation for responses. This preparation may involve several mechanisms. First, gene transcription always remains in a state of preparation and storage, with over 80% of *N. haitanensis* gene inventory remaining in an active transcriptional state (FPKM > 0; >9,000 genes) across treatments, even in the 5% RWC condition. Oliver et al. (2005) demonstrated that novel transcripts are not recruited by protein synthetic machinery during bryophyte drying and that protein synthesis is rapidly lost. This result contrasts significantly with those in the present study. Second, ribosomal function and ribosomal biogenesis remain in a transcriptional readiness state to initiate protein synthesis, with the expression of genes involved in these pathways continually increasing from hydration to 5% RWC (fig. 4 and supplementary fig. 16, Supplementary Material online; *P* < 0.05). In addition, a significant increase occurred at RH-30 s. In contrast, proteomic analysis indicated that ribosomal protein expression did not actually increase during 5% RWC conditions, but instead significantly decreased (*P* < 0.05). This result may be due to short-term carbon starvation, when the synthesis of various macromolecules would have ceased during dehydration (Lallemand et al. 2019). However, ribosomal reserves could be leveraged upon rehydration, and proteins would be quickly synthesized to restore their levels. Huang et al. (2021) also noticed the importance of the ribosome in the stress response of *N. haitanensis* to desiccation, because in the protein interaction network, multiple ribosomal relative proteins exhibited the highest connectivity values.

In addition, protein repair and correct folding after synthesis is always maintained, as indicated by the massive expansion of Hsp90 genes in the genome and by the tight transcriptional regulation of several Hsp genes via heat shock transcription factors (Hsf). Hsfs also interact with genes including the cell division control protein 48 gene (CDC48), which regulates the biogenesis of the 60S ribosomal subunit (Kressler et al. 2010); the nuclear valosin-containing protein-like (NVL) gene involved in pre-rRNA processing (Nagahama et al. 2004); mitogen-activated protein kinase (MAPK) genes (Hamel et al. 2006); and the target of rapamycin (TOR) protein-coding genes involved in activating transcription, protein synthesis, and ribosomal biogenesis (Martin et al. 2006). Some of the aforementioned genes were upregulated under dehydration or rehydration conditions, including Hsp90 genes (supplementary fig. 17, Supplementary Material online, *P* < 0.05). In addition, genes involved in DNA mismatch repair, base excision repair, and nucleotide excision repair were also upregulated during desiccation (supplementary fig. 18, Supplementary Material online).

Some proteins play primary protective roles in desiccation-tolerant plants or green algae, such as the late embryogenesis abundant (LEA) protein family, which will expand and accumulate to high quantities during dehydration (Lüttge et al. 2011; VanBuren et al. 2018). LEA proteins were not found in the genome of *N. haitanensis*, indicating that in the early eukaryotic red algae stage, LEA proteins have not yet evolved or functioned as part of the desiccation-tolerant strategy.

## Conclusions

In this study, an algal genome was isolated and completed along with those of complex epiphytic bacteria using Meta-Hi-C sequencing methods. These methods provide an ideal solution for future genome assembly of impure culture samples and help to unravel the complex relationships between algae and bacteria. Genome structure analysis of *N. haitanensis* indicated that it exhibited complex functions and evolutionary histories that might be driven by its specific lifestyle and ecology. Integration of genes from bacteria apparently helped compensate for the extensive gene loss in the red algal genomic background that was originally simplified in its ancient ancestor.

Multiomics analysis indicated overall that intertidal desiccation resistance and subsequent rapid recovery are not simple adaptations. The limited time intervals of desiccation that occur each day allow cells to make a series of preparations for recovery and repair upon dehydration. To achieve this, *N. haitanensis* immediately shuts down its photosynthetic system upon water loss to reduce photo-damage and reduce energy consumption from carbon assimilation. Furthermore, cells increase degradation activities and slow the synthesis of macromolecules. Stable mRNA pools are maintained throughout the process, and ribosomal biogenesis and ribosomal genes remain active. In addition, heat shock elements and repair mechanisms ensure the continual synthesis of proteins, along with the stability of DNA and RNA. Moreover, SAM-methyltransfer activities cleverly combine the polyamine and GSH-AA-NADPH antioxidant systems to effectively reverse inevitable oxidative damage and avoid cell death by strengthening the entire cellular antioxidant system. These unique adaptations can achieve a positive balance during a narrow window of opportunity that occurs as a result of the episodic nature of dehydration events. Consequently, the shut down-prepare-protect-repair-recovery scheme is a systematic, effective, and evolutionarily unique strategy allowing *N. haitanensis* to optimize survival in the intertidal zone and might be a common adaptive feature of intertidal algae.

## Materials and Methods

### Thalli Preparation for Genomic Analysis

*N.* *haitanensis* thalli were collected from a coastal farm at Yinzhou, Xiangshan Harbor (121.85°E, 29.66°N) within the Zhejiang province of China in October 2016. Thalli were rinsed with sterilized seawater and treated with an antibiotic cocktail (300 mg/l ampicillin, 100 mg/l kanamycin, and 100 mg/l gentamycin) for 18 h. An additional antibiotic treatment was performed using a mixture of 50 mg/l chloramphenicol, 200 mg/l cefotaxime, and 50 mg/l oleandomycin for 4 days (Gu et al. 2020). Subsequent procedures were all conducted under strict aseptic conditions.

Protoplast preparations were produced by enzymatically isolating single somatic cells from the thalli (Xu et al. 2007). Conchocelis were obtained after spontaneous haploid doubling and then were cultured in sterile medium at 20 ± 1 °C with 36 μmol photons·m^−2^·s^−1^ illumination over light: dark cycles of 12 h: 12 h until enough biomass was present to collect DNA.

### Experimental Design of Desiccation and Rehydration Treatment

To conduct desiccation and rehydration experiments, thalli collected from the same location were immersed in sea water and brought to the laboratory within 2 h. Thalli were bred in sterile seawater for 1 day before treatment. The whole experiment was conducted while incubating thalli at 20–22 °C with 40 μmol· m^−2^·s^−1^ illumination (12 h: 12 h, light: dark cycle), humidity of 70 ± 5%, and a constant flow of ambient air passed at a rate of 1.5 m/s in an air chamber. Healthy thalli were blotted to remove excess external water, weighed, and hung on strings to simulate drying stress. Continuous dehydration and rehydration simulations were then conducted. Thalli were taken at different time points for microscopic analysis and weighing (*n* = 10).

The RWC of thalli was calculated using the formula: RWC (%) = (F_w_ − D_w_)/(FT_w_ − D_w_) ×100, where F_w_ is the fresh weight of thalli after dehydration for a specific amount of time, D_w_ represents the dry weight after incubation at 80 °C for 6 h, and FT_w_ is the fresh thalli weight (Xu et al. 2018).

Thalli with various water content levels were evaluated in the following treatment groups: HD (hydration), 20% RWC, 5% RWC, RH (rehydration)-30 s, RH-5 min, RH-1 h, and RH-12 h. Thalli were collected for the corresponding groups at the specified times and immediately frozen in liquid nitrogen, with some thalli subsets being used for proteomic and transcriptomic analyses and others being ground to a fine powder and stored at −80 °C for metabolomic and metabolite analysis. In addition, the thalli from the HD, 40% RWC, 5% RWC, RH-30 s, and RH-12 h groups were collected for additional electron microscopy and staining observations.

### Genomic DNA Isolation, Library Construction, and Sequencing

Genomic DNA was extracted from *N. haitanesis* conchocelis using the cetyltrimethylammonium bromide (CTAB) method (Rogers and Bendich 1989) and DNA fragments greater than 15 kb were selected using BluePippin (Sage Science, Beverly, MA, USA). The PacBio library was prepared using the SMRTbell Template Prep Kit-SPv3 (Pacific Biosciences, Menlo Park, CA, USA) and sequenced on the PacBio Sequel system. The Illumina sequencing library was constructed using the NEBNext Ultra II DNA Library Prep Kit for Illumina (New England Biolabs, Ipswich, MA, USA) and sequenced on the Illumina HiSeq system.

### Hi-C Library Construction and Sequencing

Hi-C library construction was conducted as described previously (Yin et al. 2018), with some modifications, and as described in detail in Supplementary Material online. Proximity-ligated DNA was purified using a DNeasy Plant Mini Kit (Qiagen, Hilden, Germany). The purified DNA was sheared to a length of ∼400 bp using a Covaris M220 instrument (Covaris). Hi-C ligated junctions were then captured by Dynabeads MyOne Streptavidin C1 (ThermoFisher, Waltham, MA, USA). The Hi-C sequencing library was prepared using a NEBNext Ultra II DNA library Prep Kit for Illumina (New England Biolabs). Fragments between 400 and 600 bp were paired-end sequenced on an Illumina HiSeq platform.

### De Novo Genome Assembly and Hi-C Scaffolding

De novo assembly was conducted with the PacBio sequencing data using the FALCON assembler with seed_coverage = 55 and length_cutoff_pr = 5,000 (Walker et al. 2014). The assembly was error corrected using arrow and pilon v1.22. Hi-C sequencing reads were mapped to the assembly using the BWA aligner (v 0.7.17). Reads classified as self-ligation, nonligation, or otherwise invalid reads were filtered. The contigs were clustered into scaffolds using LACHESIS (v.c23474f). The order and orientation of the contigs were determined using Juicebox (v.1.11.08).

### Genome Annotation

Tandem Repeat Finder v.4.0959 was used to identify tandem repeats. RepeatMasker was used to annotate the repetitive elements based on the de novo library from RepeatModeler (http://www.repeatmasker.org/), LTR_FINDER, and the Repbase program (Chen 2004). Protein-coding gene annotation utilized ab initio-, homology-, RNA-sequencing-, and Iso-seq-based methods. Details for each annotation method are described in the Supplementary Material online. A consensus gene set from each source was integrated together using MAKER.

### Comparative Genomics Analyses

Protein-coding sequences (taken as the longest transcript for each gene) from *N. haitanensis* and ten other species were used to conduct gene family cluster analysis. Pairwise alignments were first constructed for all proteins using BLASTp with an *e*-value threshold of 1e^−5^ used to identify paralogs. OrthoMCL was then used with a main inflation value of 1.5 to cluster sequences into protein families. Syntenic relationships for Lox and CA gene families were visualized using the jcvi package.

A phylogenetic reconstruction of the 11 species was produced using protein sequences from single-copy genes that were first aligned using MUSCLE. The phylogenetic analysis was conducted using RAxML with the PROTGAMMAAUTO amino acid substitution model (Stamatakis 2014). Divergence times were then estimated for all species pairs using the r8s package with the phylogenetic tree and molecular clock data from the TimeTree database used as input. Divergence times for all pairs of species in the phylogenetic tree were estimated using the MCMCtree module within the PAML software package (Yang 1997).

Gene family expansion and contraction identified using CAFÉ and gene families with conditional *P* values < 0.05 were considered to have an accelerated rate of gene gain or loss (supplementary data 10, Supplementary Material online). The expanded and contracted gene families in *N. haitanensis* (*P* ≤ 0.05) were analyzed for KEGG pathway enrichment using hypergeometric test algorithms.

For synteny analysis, the *N. haitanensis* genome was aligned against the genomes of *P. haitanensis* PH40, *N. yezoensis*, and *P. umbilicalis* using Mummer (v.4.0.0beta2) and the aligned blocks were visualized using Circos.

### Identification of HGT Events

Putative HGT events were identified based on AI index (Gladyshev et al. 2008; Fan et al. 2020) and manual inspection of the phylogenetic trees. Detailed AI calculation is described in the Supplementary Material online. Candidate horizontal transferred genes were classified into three categories according to their AI scores and phylogenetic tree inspection. Genes with AI > 10 and that showed closer clustering to bacterial genes were considered to be “strong” candidates; otherwise, the gene was considered to be a “partial” candidate. Genes with AI <= 10 were considered to be “weak” candidates.

### RNA-Seq Library Construction and Sequencing

Thalli from different treatment groups were collected and immediately frozen in liquid nitrogen for subsequent RNA extraction using a Plant RNA extraction Kit (R6827, OMEGA). RNA-seq libraries were prepared using the Illumina mRNA-seq Library Preparation kit (Illumina) and sequenced on the Illumina HiSeq platform.

### RNA-Seq Data Analysis

After filtering low quality reads, the clean reads were mapped to the *N. haitanensis* genome assembly using HISAT2. The StringTie program (Pertea et al. 2015) was used to predict new transcripts, and these were combined with genome annotations to obtain the final transcriptome set. Reads were mapped to the transcriptome data set using bowtie2 and were quantified using RSEM. Correlations between thalli and replicates were examined using PCA. DEseq2 (v.1.22.2) was used to identify DEGs that were defined as those with |log_2_(fold change)| >1 and FDR significance score (*P*_adj_) < 0.05. KEGG annotation enrichment was performed on the DEGs using hypergeometric tests. Protein–protein interactions of the DEGs were predicted using the string database (http://string-db.org/).

### PacBio Iso-Seq Library Construction, Sequencing, and Analysis

Full-length cDNA was prepared using a SMARTer PCR cDNA Synthesis Kit (Takara Biotechnology) and SMRT bell libraries were constructed using the Pacific Biosciences DNA Template Prep Kit 2.0 (Pacific Biosciences). Library quantification and size distributions were evaluated using Qubit (Life Technologies) and a Bioanalyzer 2100 system (Agilent Technologies). SMRT sequencing was performed on a PacBio Sequel II platform. Full-length nonchimeric (FLNC) reads were obtained using the SMRT analysis software suite (https://www.pacb.com/support/software-downloads). Error correction of FLNC reads with clean Illumina reads was performed using Proovread (v. 2.12) using the default parameters. FLNC reads mapped to the *N. haitanensis* genome using GMAP (v. 2019-06-10; http://research-pub.gene.com/gmap/) (Wu and Watanabe 2005).

Transcripts with identical splice junctions were collapsed into one isoform. Isoforms that overlapped by at least 20% of their length on the same strand were considered to be from the same gene locus. Alternative splice events were classified and characterized by comparing different isoforms of the same gene loci with the Asprofile program (Florea et al. 2013).

### Phylogenetic Analyses

Evolutionary histories were inferred using neighbor-joining methods (Saitou and Nei 1987). Support for clade-level groups of taxa was determined based on bootstrap replicates (1,000 replicates). Evolutionary distances were calculated using the Poisson correction method (Zuckerkandl and Pauling 1965). All ambiguous positions were removed for each sequence pair before phylogenetic analyses. Phylogenetic analyses were conducted using the MEGA7 software suite.

### Determination of Photosynthetic Parameters

Photosynthetic activity (measured by *Fv/Fm*, NPQ, *qP*) of *N. haitanensis* under dehydration and rehydration conditions were recorded using a MAXI IMAGING-PAM system (Walz, Effeltrich, Germany) (*n* = 3). Phycobiliprotein measurements were conducted as described previously (Kursar et al. 1983).

### Analysis of Cellular Viability Based on Evans Blue and TUNEL Staining

Thalli were dyed with 0.5% Evans blue dye (SolarBio, Beijing, China) for 10 min in the dark. Stained thalli were then rinsed with sea water to remove excess dye and stained cells were visualized microscopically (ECLIPSE Ti-U, Nikon, Tokyo, Japan). In addition, thalli were fixed with 4% paraformaldehyde for 30 min and washed twice with sterile seawater. PBS containing 0.5% Triton X-100 was added and incubated at room temperature for 5 min. Thalli were washed and 50 μl of TUNEL solution (Beyotime, Shanghai, China) was added, followed by incubation at 37 °C for 60 min and then washing with sterile seawater in triplicate. Positive cells were visualized using Nikon Ti-U microscopy.

### Ultrastructural Studies

Scanning electron microscopy (SEM) was conducted by fixing the thalli in 3% glutaraldehyde in 0.1 M phosphate buffer (pH 7.4) for more than 4 h, followed by post-fixing with 1% OsO_4_ in phosphate buffer for 1–2 h. Thalli were then dehydrated using a gradient of ethanol concentrations, freeze dried, mounted on aluminum stubs, sputter coated with gold palladium, and analyzed using SEM (S-3400, Hitachi, Tokyo, Japan). To avoid cell wall expansion during aqueous fixation, desiccated thalli were directly subjected to critical-point drying without fixation or ethanol treatments.

Thalli for TEM were processed by fixing sliced tissues and dehydrating them in the same manner as SEM. The specimens were then infiltrated with a mixture of absolute acetone and embedded in epoxy resin, followed by sectioning with a LEICA EM UC7 ultratome (Leica, Wetzlar, Germany). Sections were then stained with 1% uranyl acetate and 1% lead citrate and examined using a TEM (H-7650, Hitachi, Japan).

### Metabolite Measurements

Carotenoid and chlorophyll extraction and detection were conducted based on previously described methods (Li et al. 2021). Quantitative analyses were achieved using the calibration curves of 20 types of carotenoids and chlorophyll (Sigma-Aldrich, St. Louis, MO, USA).

The isolation and detection of free PUFAs were performed as described previously (Chen et al. 2018) and membrane lipid analysis was conducted as described previously (Li et al. 2014). Detailed information is included in the Supplementary Material online.

### Metabolomic and Proteomics Analyses

Metabolite profiles were determined using GC–MS and LC–MS at BioNovoGene Co., Ltd. (Suzhou, China). Metabolomic assays were conducted on thalli comprising eight biological replicates. Metabolite profiling, data analysis, and routine bioinformatics analysis was then conducted using a previously described GC–MS protocol (Lisec et al. 2006). LC–MS was also performed as described previously (Tohge and Fernie 2010).

Protein extraction and quantification were conducted as described previously (Cangelosi et al. 2019). The proteomics assays for each seaweed group included eight biological replicates. Proteomic analyses were conducted at BioNovoGene Biotech (Suzhou, China) and included gel electrophoresis, sample preparation, nano-LC-ESI-MS/MS analysis, database comparisons, data label-free protein quantification, and routine bioinformatics analysis (Liu et al. 2019). Detailed information is included in the Supplementary Material online.

### Data Normalization and Statistical Analyses

When the data of three omics was subjected to multivariate data analysis, we identified discriminating classes of samples using a statistically significant threshold of Student’s *t*-test analysis (*P* ≤ 0.05). PCA analyses were performed using the SIMCA-P software package (https://www.sartorius.com/en/products/process-analytical-technology/data-analytics-software/mvda-software/simca) and the ropls package for R (www.r-project.org). A heatmap was constructed using Euclidian distances and complete linkage clustering with the heatmap package for R. The TCseq and Cairo packages of R were used for fuzzy C means clustering of normalized DEGs, differentially expressed proteins, and differentially abundant metabolites. Differentially expressed transcripts, proteins, or metabolites for each cluster or comparison group were then evaluated for enrichment of pathways in the KEGG (www.kegg.jp) or MetaboAnalyst 4.0 (www.metaboanalyst.ca) databases. Statistical significance (*P* values) for each pathway were obtained using the hypergeometric test algorithm. The abundances of pathways with *P* < 0.05 were visualized with a heatmap constructed with TBTools (https://github.com/CJ-Chen/TBtools/releases). Univariate analysis of variance (ANOVA) tests was used to determine the significance of differences in relative contents between different groups.

Except for metabolomics data, we measured the other metabolites using three biological replicates. Statistical analyses were performed using the SPSS software program (v.16.0; SPSS Inc., Chicago, IL, USA). The results are presented as mean ± standard deviation (SD) and statistical significance was evaluated based on one-way ANOVA tests; *P* values < 0.05 were considered statistically significant.

## Supplementary Material

Supplementary data are available at *Molecular Biology and Evolution Online*.

## Supplementary Material

msab315_Supplementary_Data
