# Supplementary material for: Insights into the Ancient Adaptation to Intertidal Environments by Red Algae Based on a Genomic and Multiomics Investigation of Neoporphyra haitanensis
Source: Mol Biol Evol. 2021 Nov 3;39(1):msab315. doi: 10.1093/molbev/msab315 (PMC8752119; doi:10.1093/molbev/msab315)
Supplement: msab315_Supplementary_Data [file msab315_supplementary_data.zip › Supplementary Material.pdf]

Insights into the Ancient Adaptation to Intertidal Environments by Red Algae Based on a Genomic and Multi-Omics Investigation of *Neoporphyra haitanensis*

Haimin Chen,[1,2]† Jeffrey Shih-Chieh Chu,[3]† Juanjuan Chen,[1]† Qijun Luo,[4] Huan Wang,[3] Rui Lu,[3] Zhujun Zhu,[2] Gaigai Yuan,[3] Xinxin Yi,[3] Youzhi Mao,[3] Caiping Lu,[4] Zekai Wang,[4] Denghui Gu,[4] Zhen Jin,[4] Caixia Zhang,[4] Ziyu Weng,[4] Shuang Li,[5] Xiaojun Yan,[2]* Rui Yang[1]*

[1]State Key Laboratory for Managing Biotic and Chemical Threats to the Quality and Safety of Agro-products, Ningbo University, Ningbo, Zhejiang 315211, China.

[2]Ningbo Institute of Oceanography, Ningbo, Zhejiang 315832, China.

[3]Wuhan Frasergen Bioinformatics, Wuhan, Hubei 430074, China.

[4]Collaborative Innovation Center for Zhejiang Marine High-efficiency and Healthy Aquaculture, Ningbo University, Ningbo, Zhejiang 315211, China.

[5]Ningbo Customs Technology Center, Ningbo, Zhejiang 315211, China.

[*]Corresponding authors: Xiaojun Yan, Email: yanxiaojun@nbu.edu.cn; Rui Yang, yangrui@nbu.edu.cn

Supplementary Material

Supplementary Methods

Hi-C library construction

Samples were cross-linked with 3% formaldehyde for 45 min in a vacuum at 4°C and then quenched using 0.4 M glycine. The pellets were ground using a mortar and pestle in liquid nitrogen followed by resuspension in nuclei isolation buffer. The cell suspension was then lysed and filtered. Cross-linked nuclei were treated with 0.3% SDS and neutralized with 3% Triton X-100. The resulting DNA was digested with the MboI restriction enzyme (NEB) overnight at 37°C and the reaction was stopped with heat inactivation at 65°C. Restriction fragment ends were fixed with Klenow and labeled with biotinylated cytosine nucleotides using biotin-14-dCTP (TriLINK). Blunt-end ligation was then carried out using T4 DNA ligase and incubation at 16°C overnight. After ligation, the cross-linking was reversed by incubation with proteinase K (Thermo Fisher, USA) overnight at 65°C.

Genomic assembly

Each assembled contig in the genomic assembly was also examined using BLASTn searches against the NCBI nt database (April 17, 2019, version) with a threshold *e*-value for matches set to 1e-5. Only HSPs with alignment length > 100 and alignment percent identity (PID) > 80% were retained for further analysis. If the top BLAST hit was aligned to a *Porphyra* sequence, the contig was considered as an *N. haitanensis* contig. If the top hit aligned to bacterial sequences, the contig was deemed to be a bacterial contig. If the top BLAST hit did not align to *Porphyra* sequences nor to bacterial sequences, but was associated with *N. haitanensis* contigs based on Hi-C interactions, the contig was considered an *N. haitanensis* contig. Lastly, if the top BLAST hit did not align to *Porphyra* sequences nor bacterial sequences, and the contig did not associate with *N. haitanenesis* contigs based on Hi-C data, the contig was considered to be of "unknown" origin.

Genome annotation

Tandem Repeat Finder v.4.0959 was used to identify tandem repeats, while LTR_FINDER was used to build an LTR-retrotransponson library and RepeatModeler v.1.0.10 (http://www.repeatmasker.org/RepeatModeler.html) was used to build a *de novo* repetitive element library. The two libraries were combined into a database and RepeatMasker (Tarailo-Graovac and Chen 2004) was used to annotate the repetitive elements within the database based on the *de novo* library and the Repbase program (Jurka 2000).

Protein-coding gene annotation utilized ab initio-, homology-, RNA-sequencing-, and Iso-seq-based methods. In addition, Augustus v.3.3 (Stanke et al. 2006) and Glimmer v.3.0.4 were used for *ab initio* gene prediction. For homology-based annotation, protein sequences from *Cyanidioschyzon merolae*, *Galdieria sulphuraria*, *Neopyropia yezoensis*, *Porphyra umbilicalis*, and *Chondrus crispus* were obtained from the NCBI database and aligned to the query genome using tBLASTn (Camacho et al. 2009). Exonerate (Slater and Birney, 2005) was then used to build gene structures based on the BLAST results. For RNA-seq based gene predictions, short reads were mapped to the genome using TopHat v2.1.1 (Trapnell et al. 2009) and gene structures were predicted using Cufflinks v2.2.1 (Trapnell et al. 2010). For Iso-seq based gene prediction, reads were mapped to the genome assembly using GMAP v2016-09-14 (Wu and Watanabe 2005), while TransDecoder v.4.1.0 (https://github.com/TransDecoder/TransDecoder) was used to filter the dataset to

retain high quality gene models. Lastly, a consensus gene set from each source was integrated together using MAKER (Holt and Yandell 2011).

Bacterial genome analysis.

Bacterial scaffolds were analyzed using CheckM (version 1.1.3) to estimate genomic completeness and contamination. Putative taxonomic classifications were also performed using BLASTn, GTDB-tk (version 1.6.0), and PATRIC (Davis et al. 2020) database searches. Preliminary classifications were used to identify reference genomes for comparison of each scaffold. Whole genome alignments were performed using Mummer (v.4.0.0beta2). Contigs in scaffolds were rearranged to match the order and orientation in the reference genomes. To assess how well the bacterial sequences corresponded to known bacterial genomes, the top BLASTn hit for each superscaffold was selected for whole-genome alignment between the two genomes, followed by visualization of synteny using Circos (http://circos.ca/). Gene annotations of bacterial scaffolds were performed using DRAM (Shaffer et al. 2020) and glimmer (version 3.02).

Identification of horizontal gene transfer events

To identify candidate horizontally transferred genes, we specifically examined genes of *N. haitanensis* (not including unplaced contigs or organelle contigs) using Alien Index (Gladyshev et al. 2008; Fan et al. 2020) as well as by manually inspecting the phylogenetic tree (Wang et al. 2020; Qiu et al. 2013). Protein sequences encoded by the genomes of *N. haitanensis, C. crispus, C. merolae, G. chorda, P. purpureum,* and *P. umbilicalis* were used for horizontal gene transfer (HGT) analysis. Each sequence was aligned to the Refseq database using diamond (Buchfink et al. 2014); and the top 1,000 significant hits, sorted by bit-score, were considered in the analysis. Up to 60 corresponding BLAST hit sequences were then retrieved from the database with no more than three sequences retrieved for each genus and no more than 12 sequences retrieved for each phylum. We then calculated the AI score for each query gene following previously described methods (Gladyshev et al. 2008; Fan et al. 2020). The AI score is calculated with the following formula: AI = $(\ln(bbhG + 1 \times 10^{-200}) - \ln(bbhO + 1 \times 10^{-200}))$, where bbhG is the *e*-value of the best hit of the within-group lineage, and bbhO is the *e*-value of the best hit to species outside of the group. When no significant hits were detected, the corresponding bbhG or bbhO values were set to 1. An AI score >0 indicates a better match of the query sequence in bacteria. The higher the AI score, the more similar the queries were to their bacterial homologs than to eukaryotic homologs. We identified 522 genes that showed AI scores > 0. We filtered those genes that were not residing on the chromosomes, which left 489 genes. To further categorize these candidates, those with AI scores $\leq$ 10 and residing on *N. haitanensis* chromosomes were labelled as ‘Weak’. Genes with AI scores > 10 were labeled as ‘Partial’. Genes with AI > 10 were searched in RefSeq database using Blastp and the top 1000 hits were taken to generate a multiple sequence alignment using MUSCLE (Edgar 2004). A phylogenetic tree was built for each gene cluster using FastTree (version 2.1.11) (Price et al. 2010). Finally, genes with AI score > 10 and that clustered together with bacterial genes on the phylogenetic tree were labelled as ‘Strong’. Using this strategy, we identified 267 genes as strong HGT candidates, 33 genes as partial HGT candidates, and 189 genes as weak HGT candidates.

Ultrastructural studies

Scanning electron microscopy (SEM) was conducted by fixing thalli in 3% glutaraldehyde in 0.1 M phosphate buffer (pH 7.4) for > 4 h, followed by post-fixing with 1% OsO_4 in phosphate buffer for 1–2 h. Thalli were then dehydrated using a gradient of ethanol concentrations, freeze-dried, mounted on aluminum stubs, sputter-coated with gold palladium, and finally analyzed using an SEM instrument (S-3400, Hitachi, Japan). To avoid cell wall expansion during aqueous fixation, desiccated thalli were directly subjected to critical-point drying without fixation or ethanol treatment.

Thalli subjected to transmission electron microscopy (TEM) were processed by fixing sliced tissues and dehydrating as described above for SEM analysis. The specimens were then infiltrated with a mixture of absolute acetone and embedded in epoxy resin, followed by sectioning with a LEICA EM UC7 ultratome instrument (Leica, Germany). Sections were then stained with 1% uranyl acetate and 1% lead citrate, followed by examination with a TEM instrument (H-7650, Hitachi, Japan).

Analysis of cellular viability based on Evans blue and TUNEL staining

To assess cellular viability, thalli were dyed with 0.5% Evans blue dye (SolarBio, China) for 10 min in the dark. Stained thalli were then rinsed with sea water to remove excess dye, and stained cells were microscopically visualized (ECLIPSE Ti-U, Nikon, Japan). Further, thalli were fixed with 4% paraformaldehyde for 30 min and washed twice with sterile seawater. PBS containing 0.5% Triton X-100 was added to the samples and incubated at room temperature for 5 min. Thalli were then washed and 50 μL of TUNEL solution (Beyotime, China) was added, followed by incubation at 37°C for 60 min and then washing with sterile seawater in triplicate. Cells were then visualized with a Nikon Ti-U microscope.

Carotenoid and chlorophyll measurements

To obtain carotenoid and chlorophyll measurements, 50 mg of freeze-dried thalli were extracted with 2 mL of acetone containing 0.1% butylated hydroxytoluene (BHT) to avoid oxidation. The mixture was then ultrasonicated for 30 min and placed at −20°C for 12 h. The samples were then centrifuged at 12,000 r/min (4°C) for 5 min and filtered using a 0.22 μm membrane. All extraction procedures were performed in triplicate and the data are expressed as means ± SD.

An ultra-performance liquid chromatography (UPLC) (Thermo Fisher, UltiMate 3000) coupled to a quadrupole electrostatic field orbital trap high-resolution mass spectrometer (Q-orbitrap-HRMS) (Thermo Fisher, Quadrupole-Exactive) that was equipped with an H-ESI II source were used for qualitative and quantitative analyses of carotenoids. Qualitative analysis was conducted using the high-throughput screening software package Exactfinder™ by comparing m/z values (mass error $\leq 5 \times 10^{-6}$) in MS spectra, retention times, isotopic distributions, and MS^2 spectra, which can all help obtain qualitatively accurate results and avoid false positive results. Quantitative analyses were achieved using the calibration curves of 20 types of carotenoids and chlorophyll that were constructed with concentrations on the x-axis and the corresponding peak area values on the y-axis.

Analysis of free polyunsaturated fatty acids

Frozen powdered thalli (15 mg) were ultrasonically extracted with a chloroform: methanol (2: 1) mixture three times. Then, 2 mL methanol containing 1% sulfuric acid was added to

each resultant liquid solution for esterification at 80°C for 30 min. The samples were then dried under an N_2 stream, redissolved in hexane, and analyzed using a Shimadzu QP2010 GC-MS. GC analysis was performed using a TG-FAME silica capillary column (50 m × 0.25 mm × 0.20 μm; Thermo Fisher). The temperature of the injector was 250°C, the helium carrier gas flow rate was 0.63 mL min^{-1}, and the pre-column pressure was 51.6 kPa. After injection, the oven temperature was held at 80°C for 1 min, raised to 160°C at 20°C min^{-1}, held for 1.5 min and then raised to 196°C at 3°C min^{-1}, followed by a hold for 8.5 min and an additional increase to 250°C at 20°C min^{-1}, followed by a hold for 3 min. The mass spectrometer was operated in electron compact mode with 1 kV of electron energy. Ion source and interface temperatures were 200 and 250°C, respectively. The mass range was set from m/z 50 to m/z 750.

Membrane lipid analysis

Total lipids from each sample were extracted according to previously described methods (Bligh and Dyer 1959). Lipids were then analyzed on a Thermo Fisher U3000 UHPLC using an ACQUITY UPLC BEH C18 analytical column (i.d.: 2.1 mm × 100 mm, particle size: 1.7 m, pore size: 130 Å). Mass spectrometry was performed on a Thermo Scientific™ Q Exactive hybrid quadrupole-Orbitrap mass spectrometer equipped with a HESI-II probe. The flow rate was set at 0.2 mL/min with the mobile phase A composed of acetonitrile: water (6:4, v/v) and mobile phase B composed of isopropanol: acetonitrile (9:1, v/v), both containing 0.1% formic acid and 10 mM ammonium acetate. The gradient elution was programmed as follows: 0–15 min, 60–45% A; 15.0–18.0 min, 45–35% A; 18.0–26.0 min, 35% A; 26.0–28.0 min, 35–0% A; 28.0–30.0 min, 0% A; 30.0–30.5 min, followed by a return to an initial 60% A; 30.5–40 min, 60% A.

Mass spectrometry was also performed on a Thermo Scientific™ Q Exactive hybrid quadrupole-Orbitrap mass spectrometer equipped with a HESI-II probe. The instrument was operated in a data-dependent LC-MS/MS manner in positive and negative modes, respectively. Data were acquired in a centroid mode in the range of m/z 150 to 1,500 at a resolution of 70 K, and in the high energy collisional dissociation (HCD) MS/MS mode at 17.5 K resolution. The automatic gain control target was set at $1e^6$ for MS and $2e^5$ for MS^2. The capillary voltage was set at 3.5 kV and the capillary temperature was 350°C. The sheath gas was set at 45 arb and the aux gas was set at 10 arb. MS^2 analysis was performed with the mass spectrometer with various collision energies and a ramp of 25 and 30 V in positive ion mode and 20, 24, and 28 V in negative ion mode based on different lipid types. Lipidsearch software version 4.1 (Thermo Scientific™) was used to analyze the MS data.

Metabolomic analyses

Thalli comprising eight biological replicates were used for all metabolomic assays. Extracted samples were subjected to GC-MS (GC, Agilent 7890B; MS, LECO Pegasus BT) using a DB-5MS capillary column (30 m × 250 μm, Agilent J & W Scientific, Folsom, CA, USA), and to LC-MS (HPLC Thermo Ultimate 3000 system; MS, Thermo Q Exactive) using an ACQUITY UPLC® HSS T3 column (1.8 μm, 2.1 × 150 mm, Ethylene Bridged Hybrid, Waters). Metabolites were confirmed based on their exact molecular weights, mass spectra, and retention time index values. The original GC-MS and LC-MS data were converted into mzXML format using Proteowizard software and were processed and analyzed using the XCMS software package (www.bioconductor.org) with optimized

settings. Metabolite annotation of the GC-MS data was conducted with an automatic processing and identification system (AMDIS), by referencing databases of the National Institute of Standards and Technology (NIST) and the Wiley Registry of Mass Spectral Data (Wiley Online Library). Metabolite annotation of the LC-MS data was conducted with the Compound Discoverer program and by reference to the mzCloud database (www.mzCloud.org), the Human Metabolome Database (www.hmdb.ca), the METLIN database (metlin.scripps.edu), the MassBank database (www.massbank.jp), and the LIPID MAPS database (www.lipidmaps.org), in addition to an in-house standard product database at BioNovoGene Co., Ltd. The data were scaled by autoscaling before multivariate statistical analysis. To compare data of different orders of magnitude, IS normalization of peak area was carried out for GC-MS data, and batch normalization of peak area was carried out for LC-MS data. Differentially abundant metabolites between two groups of samples were identified using a statistical significance threshold based on Variable Importance in Projection (VIP) values (VIP ≥ 1) and then further validated by Student's *t*-test analysis ($p \leq 0.05$). Metabolite correlation was assessed using Pearson correlation coefficients and these data were used to construct a network using the Cytoscape software program (www.cytoscape.org). To identify alternative metabolic pathways, differential metabolites were subjected to grouping and enrichment of metabolic pathways using the MetaboAnalyst 4.0 software program (www.metaboanalyst.ca) and the KEGG database (www.kegg.jp).

Proteomics analyses

Proteins of freeze-dried thalli were prepared by SDT lysis (50 mM NH_4HCO_3, 75 mM NaCl, 2% sodium deoxycholate) and were precipitated by acetone. The total protein of all the samples was extracted, followed by digestion with trypsin (Promega, USA) (Jorrin-Novo, 2014; Wisniewski, Zougman, Nagaraj, & Mann, 2009). The peptides were desalted and freeze-dried. Then the peptides were reconstituted with 50 µL 0.1% formic acid and analyzed using an Oribtrap Fusion Lumos MS (Thermo Scientific, USA) coupled online with a nanoscaled liquid chromatography system (Easy-nLC 1200, Thermo Fisher Scientific, USA). Chromatographic separation was equipped with a reverse-phase C18 column (ACQUITY UPLC® BEH C18, 150×2.1 mm, 1.8 µm, Waters), a peptide capture column (Acclaim PepMap C18, 100 µm×20 mm, Thermo Scientific), and a peptide analysis column (Acclaim PepMap C18, 75 µm×250 mm, Thermo Scientific). The separation of fractions was performed, and liquid chromatography-tandem mass spectrometry (LC-MS/MS) was employed to analyze the separated peptides. After quality control, the resulting MS/MS data were processed using Proteome Discoverer (version2.2 Thermo Scientific) for identification and quantitation analysis according to the database of the RNA-Seq test to predict the protein sequence library. The protein quantitative value was standardized by the median to eliminate the sample amount error caused by the sample amount and instrument operation. Student's *t*-test was used for statistical analysis. Proteins with a $p < 0.05$ and an absolute fold change ≥ 1.5 were considered significantly different.

Supplementary Figures and Figure legends

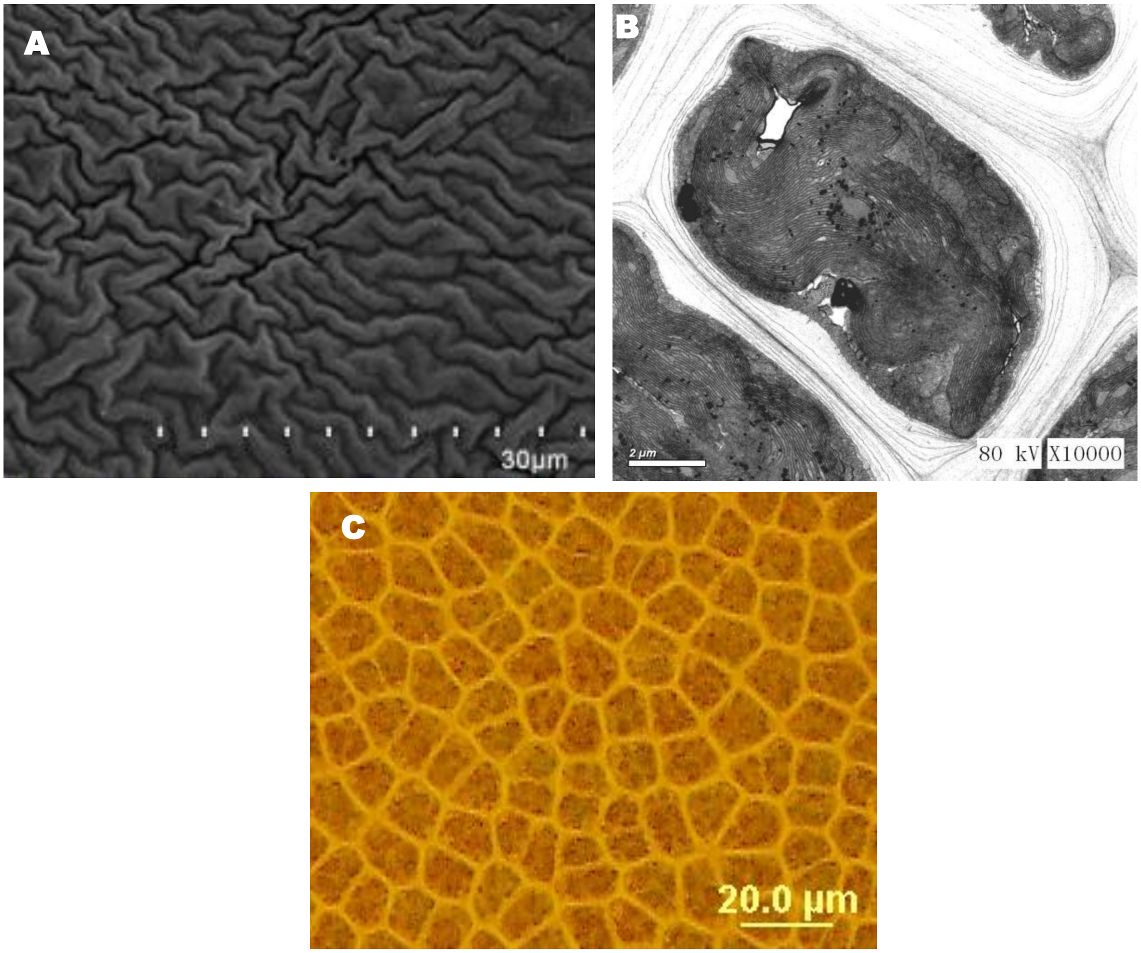

Supplementary fig. 1. *Neoporphyra haitanensis* thalli after multiple antibiotic XII treatments. Thalli were treated with an antibiotic cocktail (300 mg/L ampicillin, 100 mg/L kanamycin, and 100 mg/L gentamycin) for 18 h. A further antibiotic treatment was then performed with a mixture of 50 mg/L chloramphenicol, 200 mg/L cefotaxime, and 50 mg/L oleandomycin for 4 d. The thalli were then observed under a (A) scanning electron microscope, (B) transmission electron microscope, and (C) light field microscope.

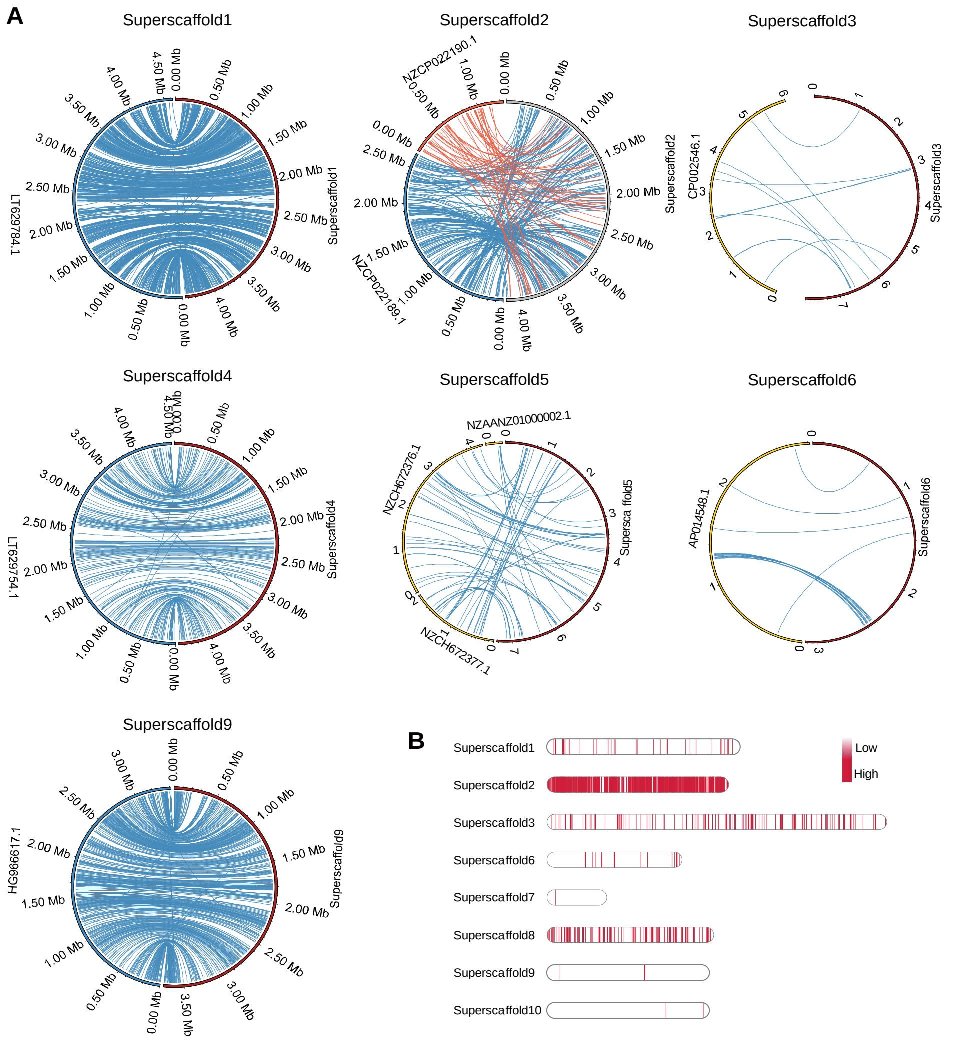

Supplementary fig. 2. Synteny analysis of epiphytic bacterial genomes and the distribution of their gene sequences on superscaffolds. (A) Synteny analysis of epiphytic bacterial superscaffolds separated from the *Neoporphyra haitanensis* genome using Hi-C assembly methods. The sequences from each superscaffold were aligned using MUMmer 4.0 (http://mummer.sourceforge.net) and circular visualizations were made with the Circos software program (http://circos.ca/). Superscaffold 1 (4.44 Mbp) was aligned to the *Stappia* sp. ES.058 genome assembly (LT629784.1), Superscaffold2 (4.19 Mbp) was aligned to the *Yangia pacifica* YSBP01 chromosome 1 and 2 complete sequences CP022189.1), Superscaffold3 (7.82 Mbp) was aligned to the *Rubinisphaera brasiliensis* DSM 5305 chromosome (CP002546.1), Superscaffold4 (4.48 Mbp) was aligned to the *Maribacter* sp. MAR_2009_60 genome assembly (LT629754.1), Superscaffold5 (7.34 Mbp) was aligned to the *Blastopirellula marina* DSM 3645 genome (GCA_000153105.1), Superscaffold6 (3.12 Mbp) was aligned to the *Nonlabens marinus* S1-08 nearly complete genome (AP014548.1), and Superscaffold9 (3.37 Mbp) was aligned to the *Candidatus* Phaeomarinobacter ectocarpi complete genome (HG966617.1). **(B)** Distribution of annotated genes on the superscaffolds. Each colored bar represents gene density over a 10 kbp window.

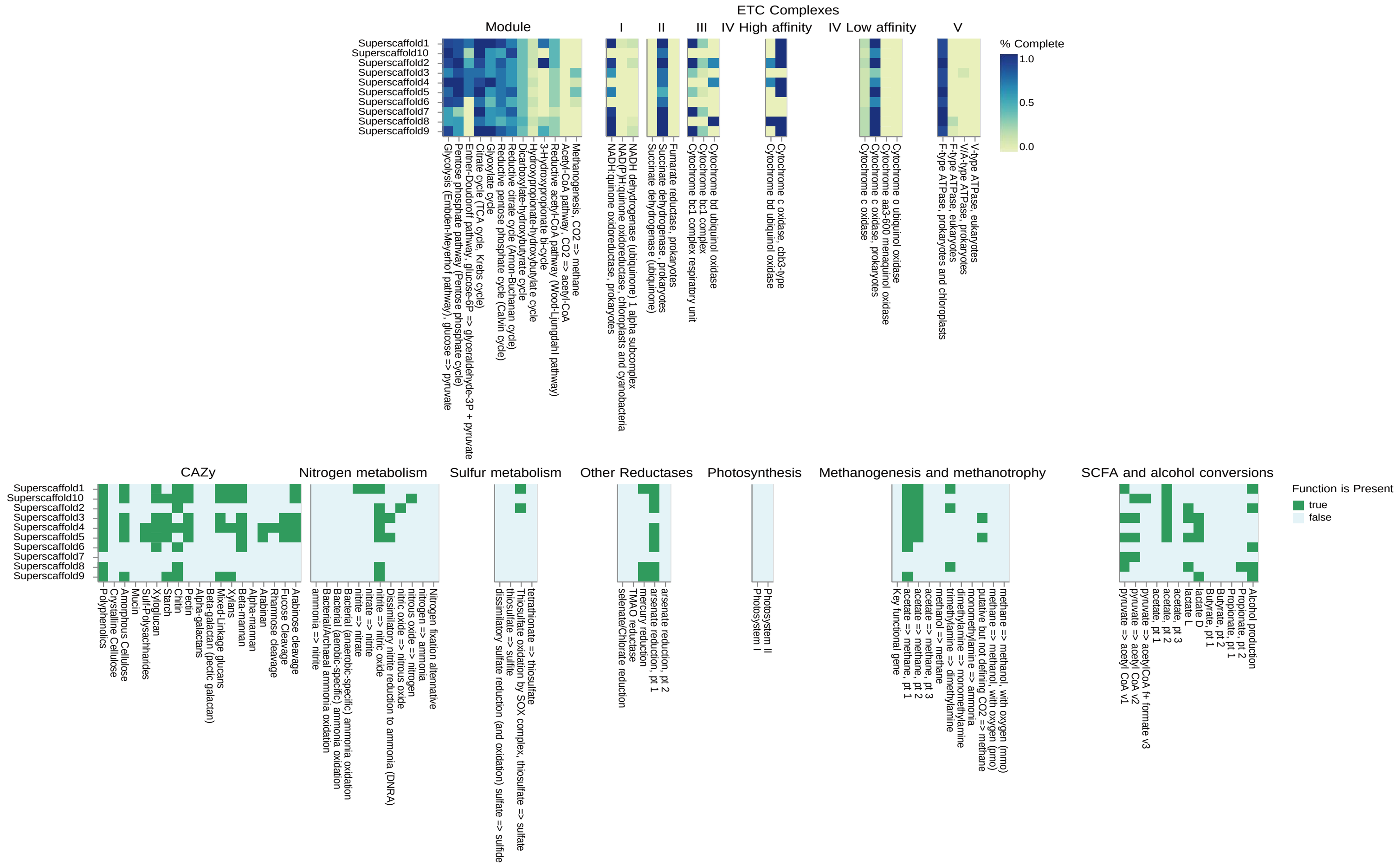

Supplementary fig. 3. Distilled and refined annotation of metabolism (DRAM) of 10 bacterial superscaffolds separated from the *Neoporphyra haitanensis* genome.

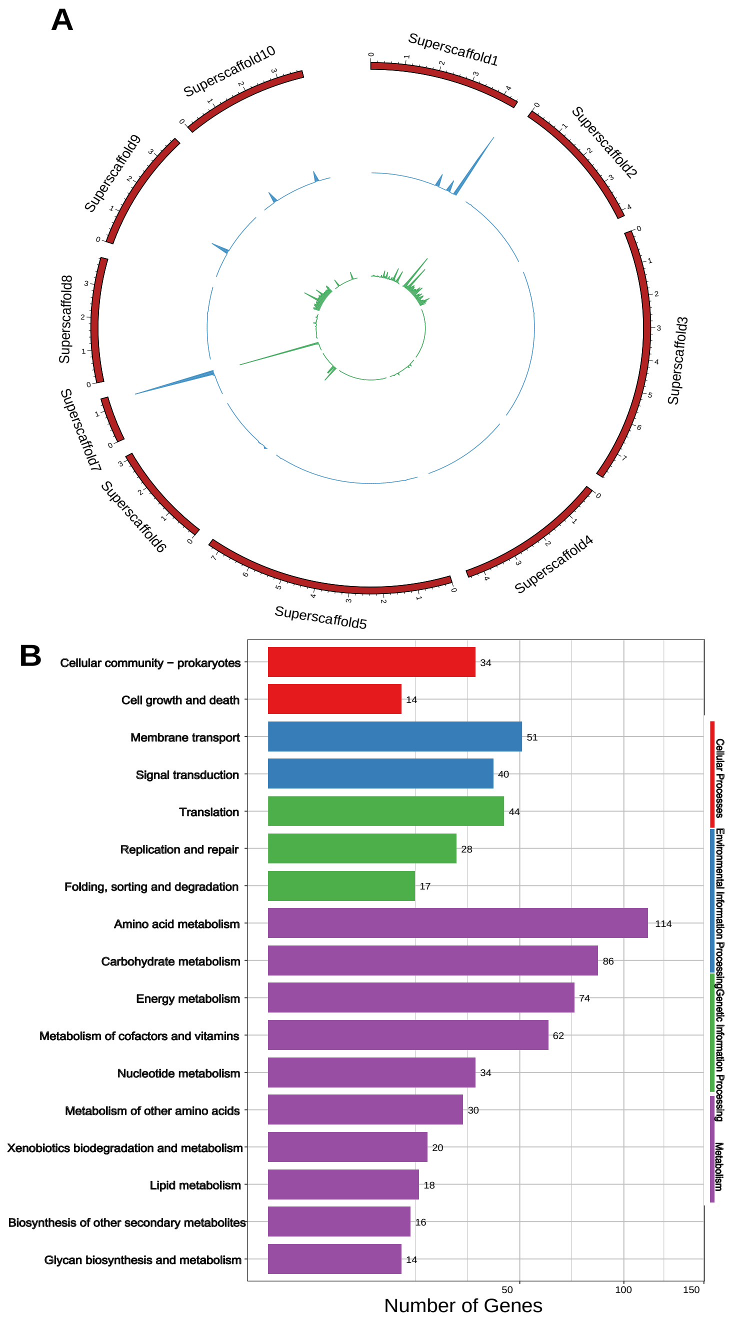

Supplementary fig. 4. Analysis of common sequences among epiphytic bacterial genomes of *Neoporphyra haitanensis* and other *Porphyra* genome assemblies. (A) Alignment depth of the *Pyropia haitanensis* PH40 subreads and the *Porphyra umbilicalis* subreads to *N. haitanensis* epiphytic bacterial genomes using minimap2 (https://github.com/lh3/minimap2). The circle graphs were drawn according to the alignment depth using the Circos software package (http://circos.ca/). The red circle represents the epiphytic bacterial genome sequence skeleton, while the blue and green circles represent the location and depth of *P. haitanensis* PH40 and *P. umbilicalis* subreads aligned to the *N. haitanensis* epiphyte sequences, respectively. **(B)** KEGG enrichment of common annotated sequences from the *N. haitanensis* epiphyte genomes with the *P. haitanensis* PH40 and *P. umbilicalis* genomes.

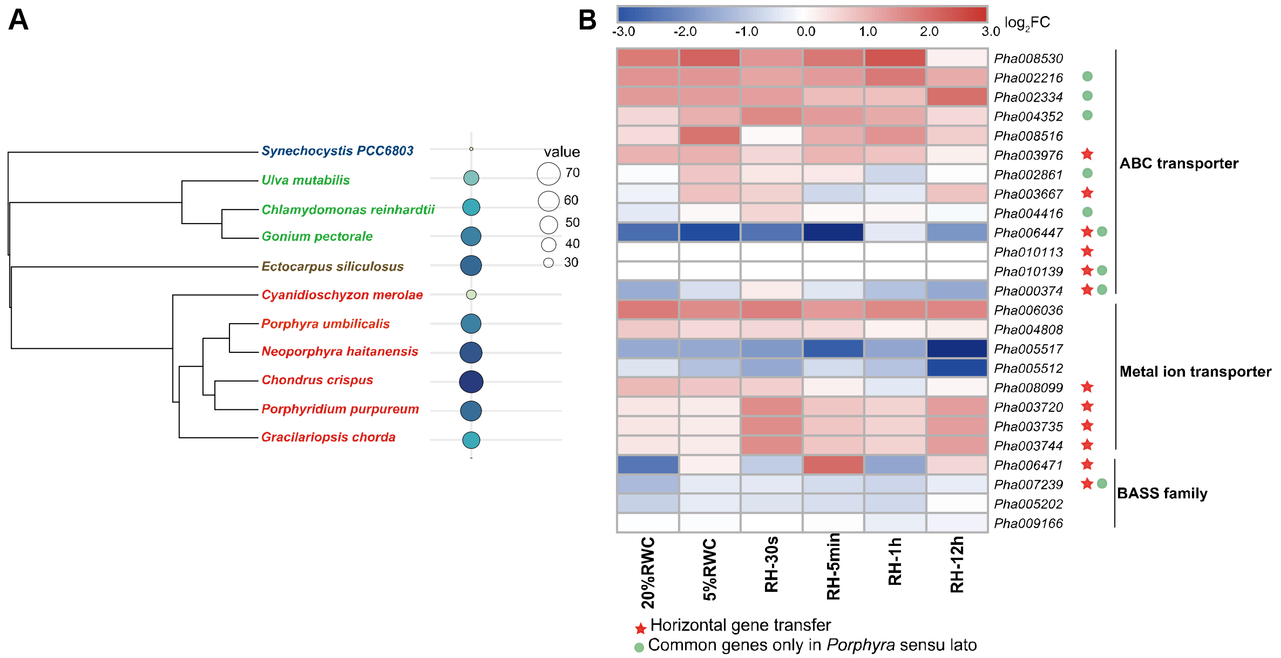

Supplementary fig. 5. Comparison of transporter genes in the *Neoporphyra haitanensis* genome. (A) Copy numbers of ATP-binding cassette (ABC) transporter genes in algal genomes. The dendrogram is based on the phylogenetic reconstruction of single-copy orthologous genes from *N. haitanensis* and other published algal genomes. Circle sizes indicate gene copy numbers. **(B)** Heatmap showing variation in gene expression levels ($\log_2$ fold-changes) of transporter-related genes under desiccation and rehydration treatments. Fold change was calculated as FPKM (x% RWC or rehydration (RH) / FPKM (hydration control).

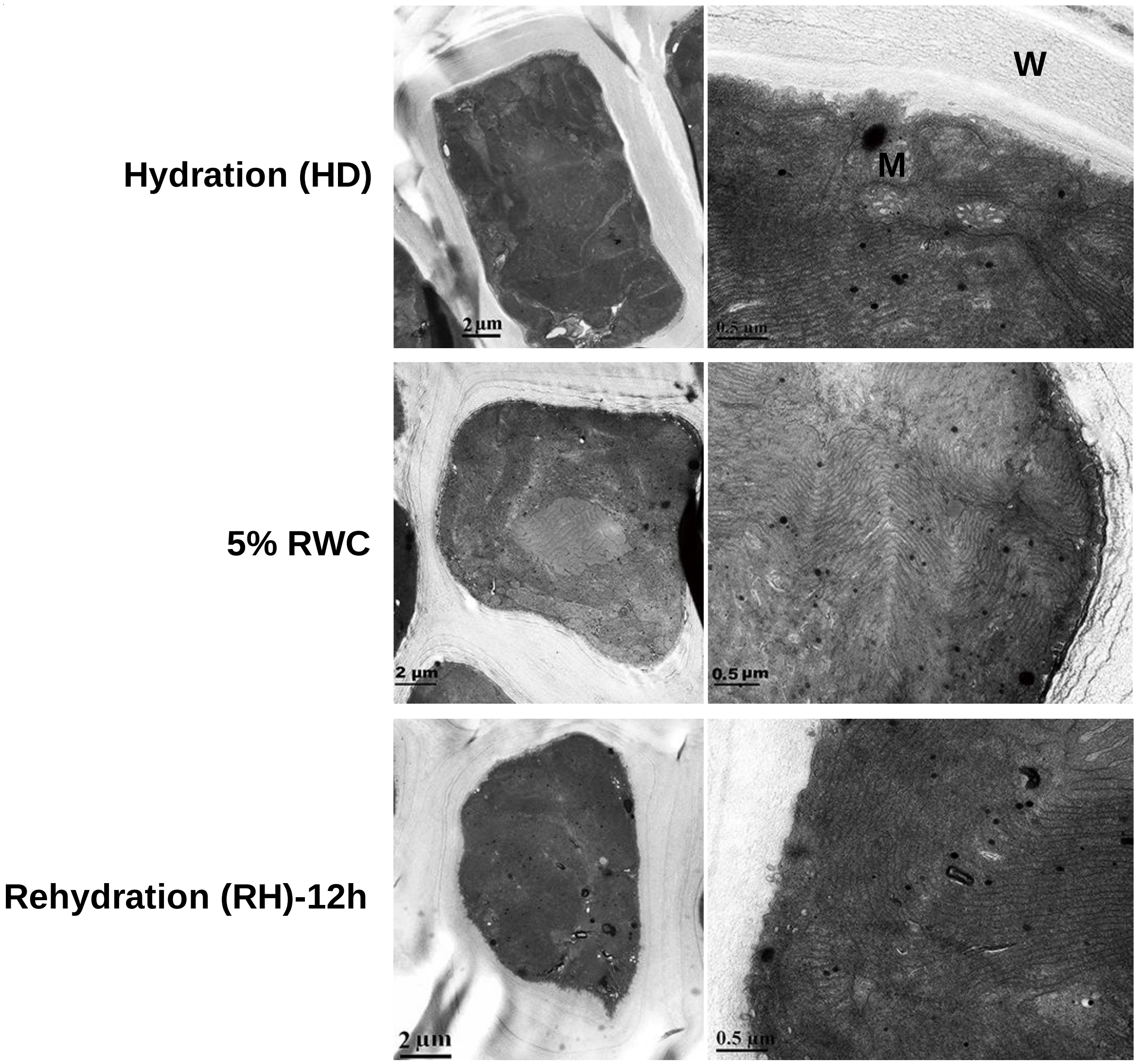

Supplementary fig. 6. Transmission electron micrographs of *Neoporphyra haitanensis* cells from the hydrated (HD, 3.33 ± 0.1 g H_2O/ dry weight), 5% RWC (0.17 ± 0.01 g H_2O/dry weight), and rehydration for 12 h (RH-12h, 2.88 ± 0.27 g H_2O/dry weight) conditions. Note the intact (T; closed double membrane layer) thylakoids in the three groups, and the wrinkled cell wall (W) of dried cells. Scale bars: 2 µm and 0.5 µm.

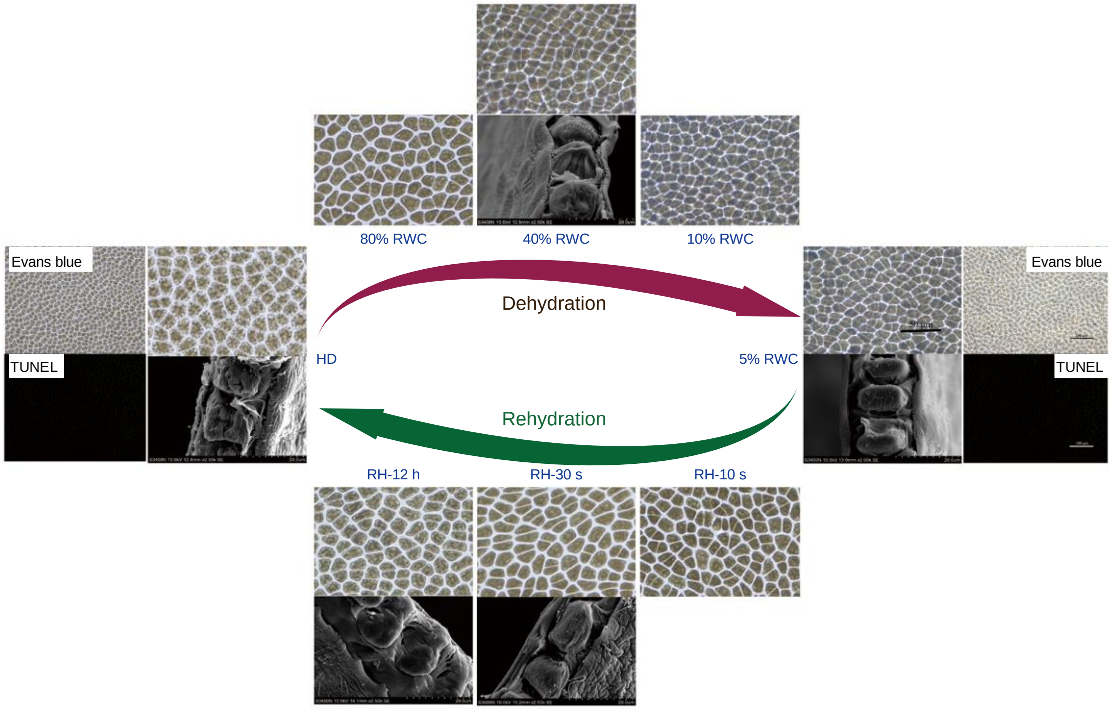

Supplementary fig. 7. Microscopic cellular morphological changes after dehydration and rehydration. The micrographs of cells under different relative water content (RWC) treatments are shown after visualization at 400x. Scanning electron micrographs of the thalli transection are shown with a scale bar = 20 µm. Evans blue and TUNEL stained cells are shown at 200x. Fresh thalli were dehydrated to 5% RWC over 30 min and rapidly rehydrated within 12 h. Thalli subjected to hydration (HD), 80% RWC, 40% RWC, 10% RWC, and rehydrated thalli (RH) after 10 s, 30 s, and 12 h were harvested for imaging and subjected to Evans blue and TUNEL staining.

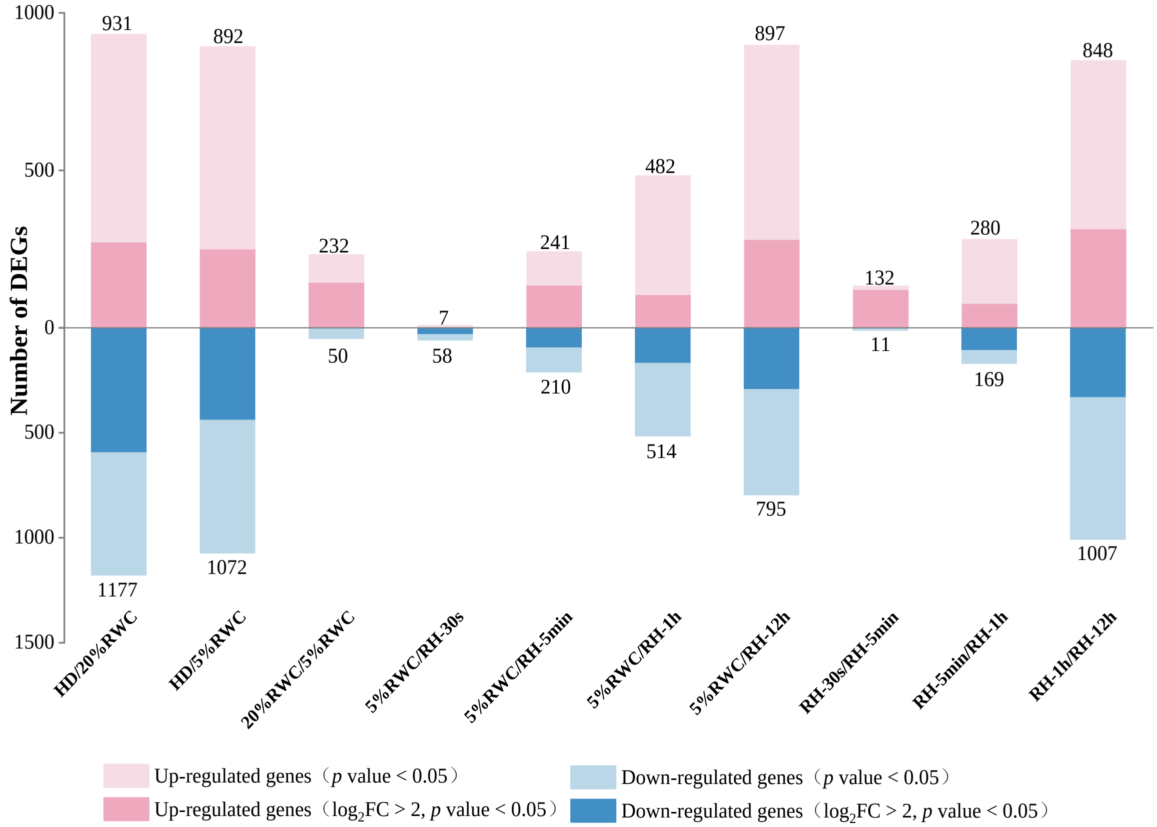

Supplementary fig. 8. Number of differentially expressed genes (DEGs). HD: hydration; RH: rehydration.

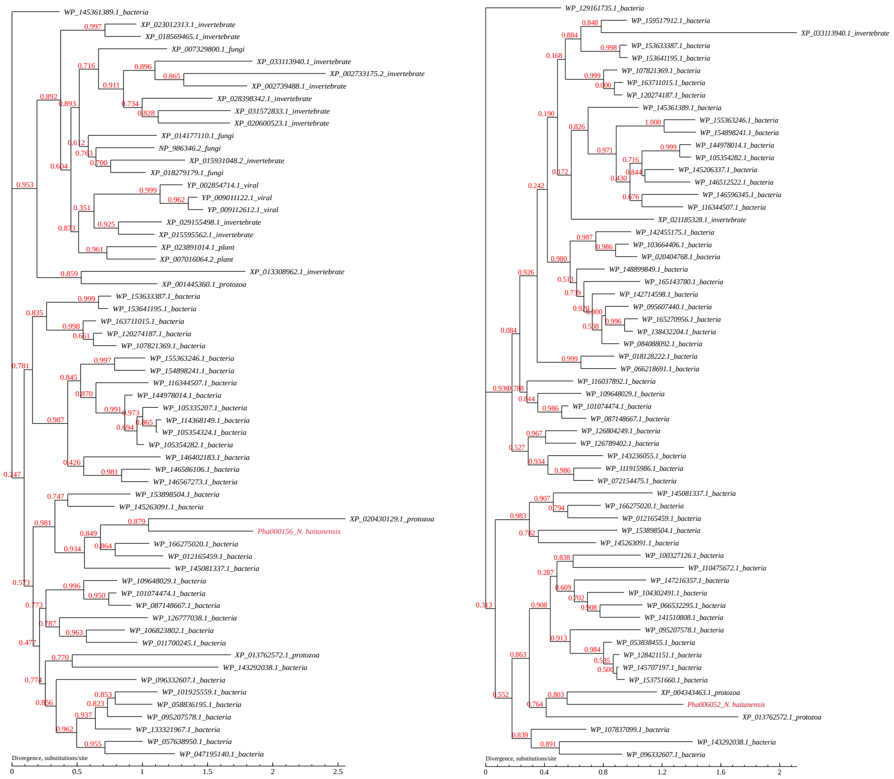

Superoxide dismutase, Cu-Zn

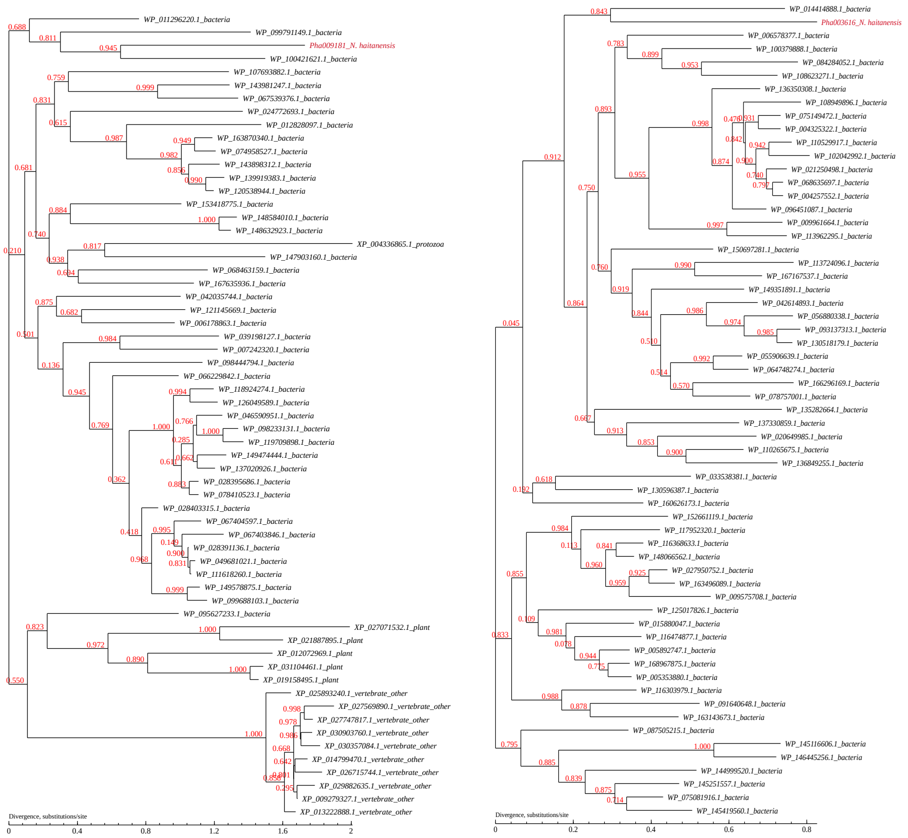

Carbonic anhydrase

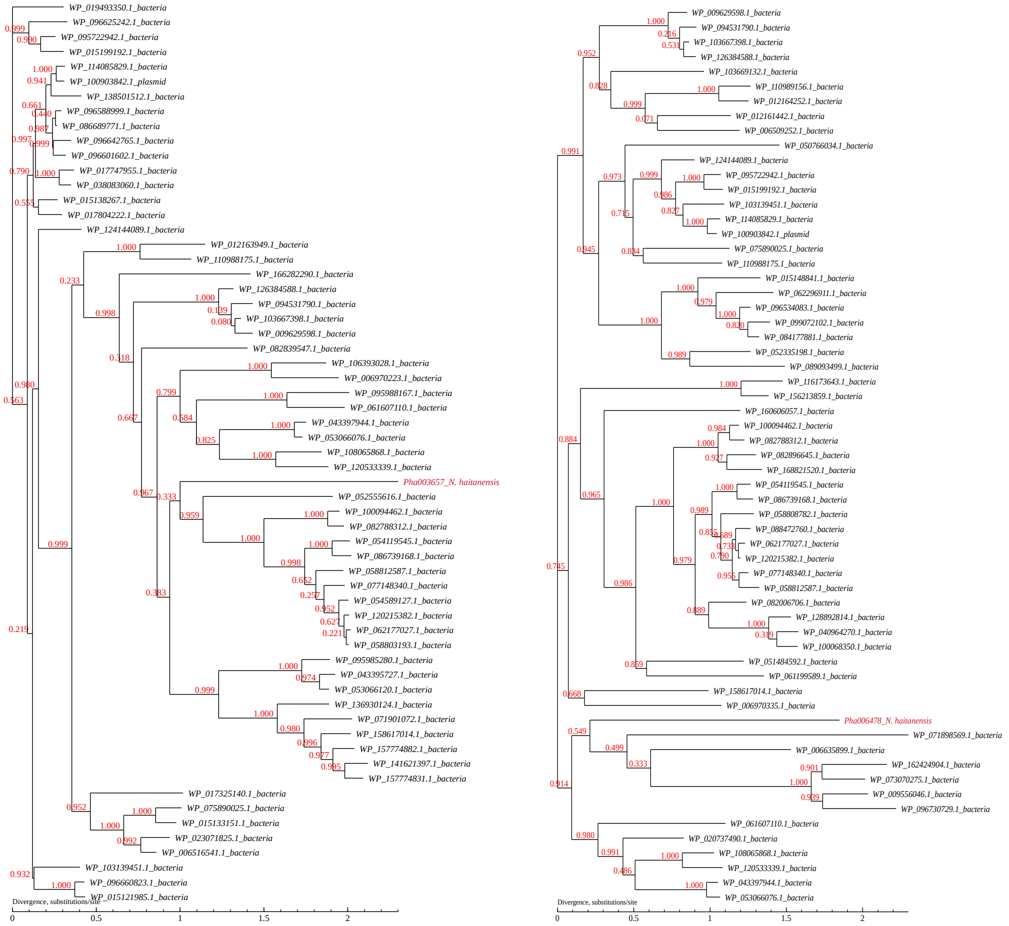

Lipoxygenase

Supplementary fig. 9. Phylogenetic trees of typical horizontal gene transfer (HGT) genes

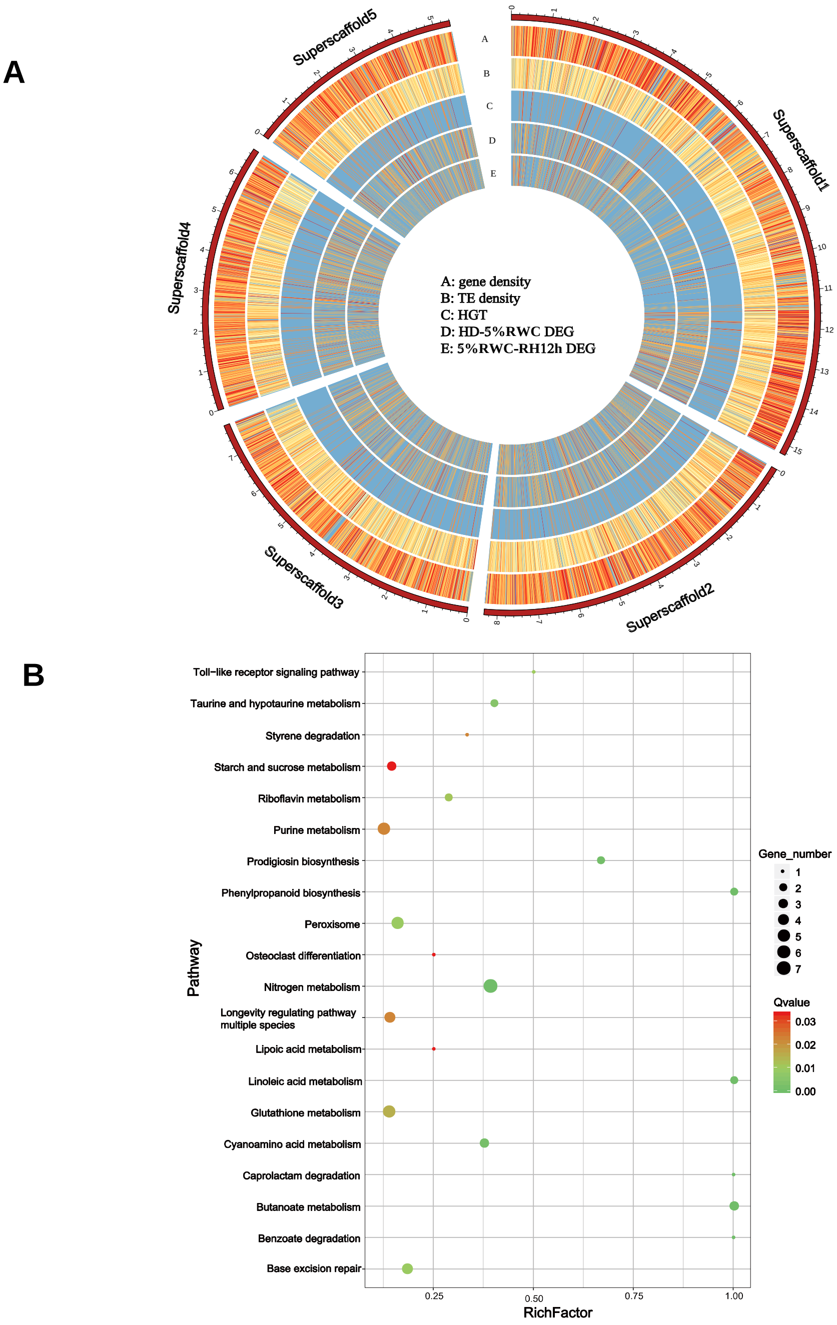

Supplementary fig. 10. Distribution of horizontal gene transfer (HGT) events into the *Neoporphyra haitanensis* genome and their associated functional enrichment. (A) Genomic profiles integrating genome structures with HGT and differentially expressed genes (DEGs). The genome map displays (from outside to inside): A. Gene density; B. Transposable element (TE) density; C. HGT; D and E. DEGs between hydrated (HD) vs. 5% RWC, and 5% vs. RH (rehydrated)-12h conditions, respectively, as identified by greater than two-fold changes in transcript abundances compared to hydrated controls ($p < 0.05$). **(B)** Functional categorization of HGT candidate genes. Biological functions were defined by the associated KEGG enrichment values.

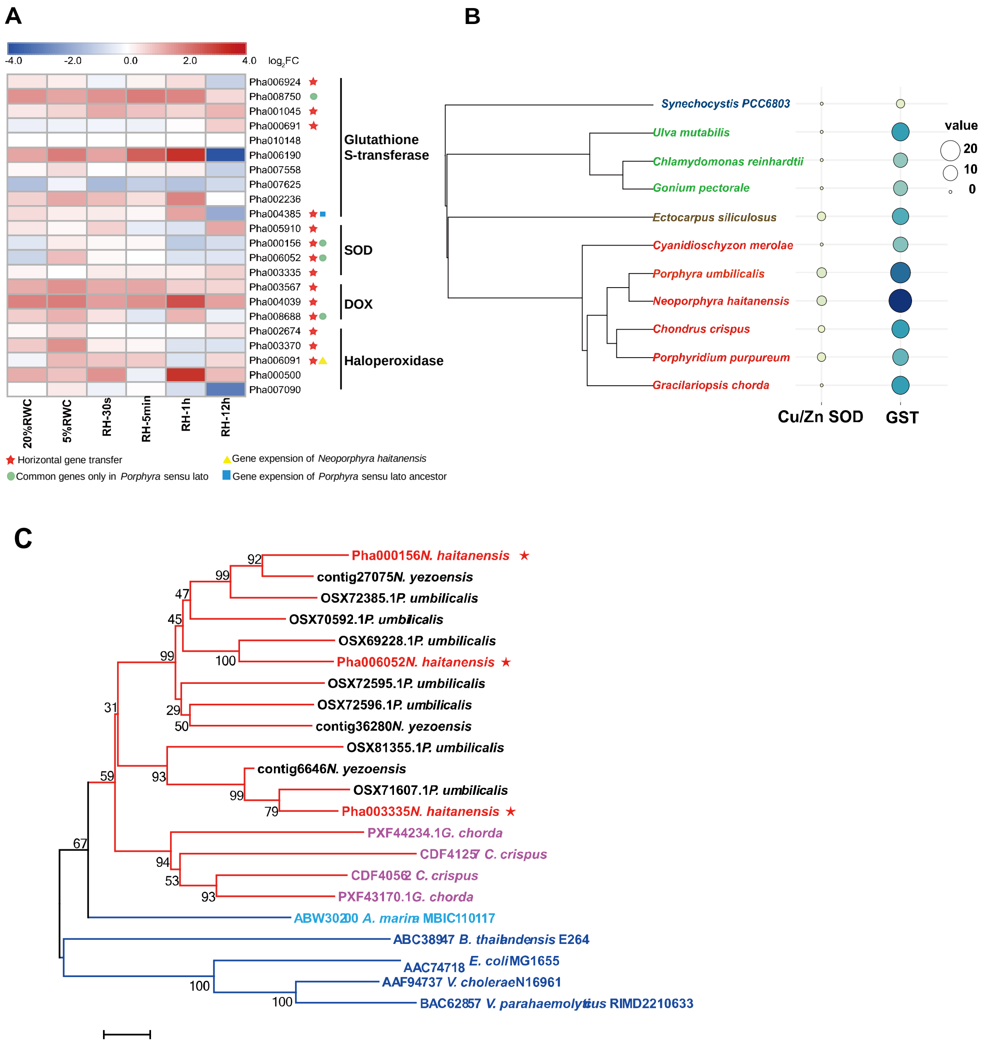

Supplementary fig. 11. Antioxidant-related genes in the *Neoporphyra haitanensis* genome. (A) Heatmap showing the transcriptional patterns of antioxidant-related genes in response to desiccation and rehydration. Data are expressed as $\log_2$ fold-change values. Fold change was calculated as FPKM (x% RWC or rehydration (RH) / FPKM (hydration control). **(B)** Copy numbers of Cu/Zn superoxide dismutase (SOD) and glutathione S-transferase (GST) genes in algal genomes. The dendrogram was generated based on the phylogenetic analysis of single-copy orthologous gene sets identified from *N. haitanensis* and the other published algal genomes. Circle sizes represent gene copy numbers. **(C)** Phylogenetic analyses of Cu/Zn SOD homolog sequences from different species. All phylogenetic trees were constructed using neighbor-joining methods in MEGA7 with 1,000 bootstraps to evaluate node support. Red branches indicate red algal genes including those from *N. haitanensis*, *Porphyra umbilicalis*, *Neopyropia yezoensis*, *Chondrus crispus*, and *Gracilariopsis chorda*. Blue branches represent homologous bacterial genes, including those from *Burkholderia thailandensis* E264, *Escherichia coli* str. K-12 substr. MG1655, *Vibrio cholerae* O1 biovar El Tor str. N16961, *Vibrio parahaemolyticus* RIMD2210633, and *Acaryochloris marina* MBIC110117. Homologs from the *N. haitanensis* genome are highlighted in red text, and suspected horizontal gene transfer (HGT) genes are highlighted with red stars.

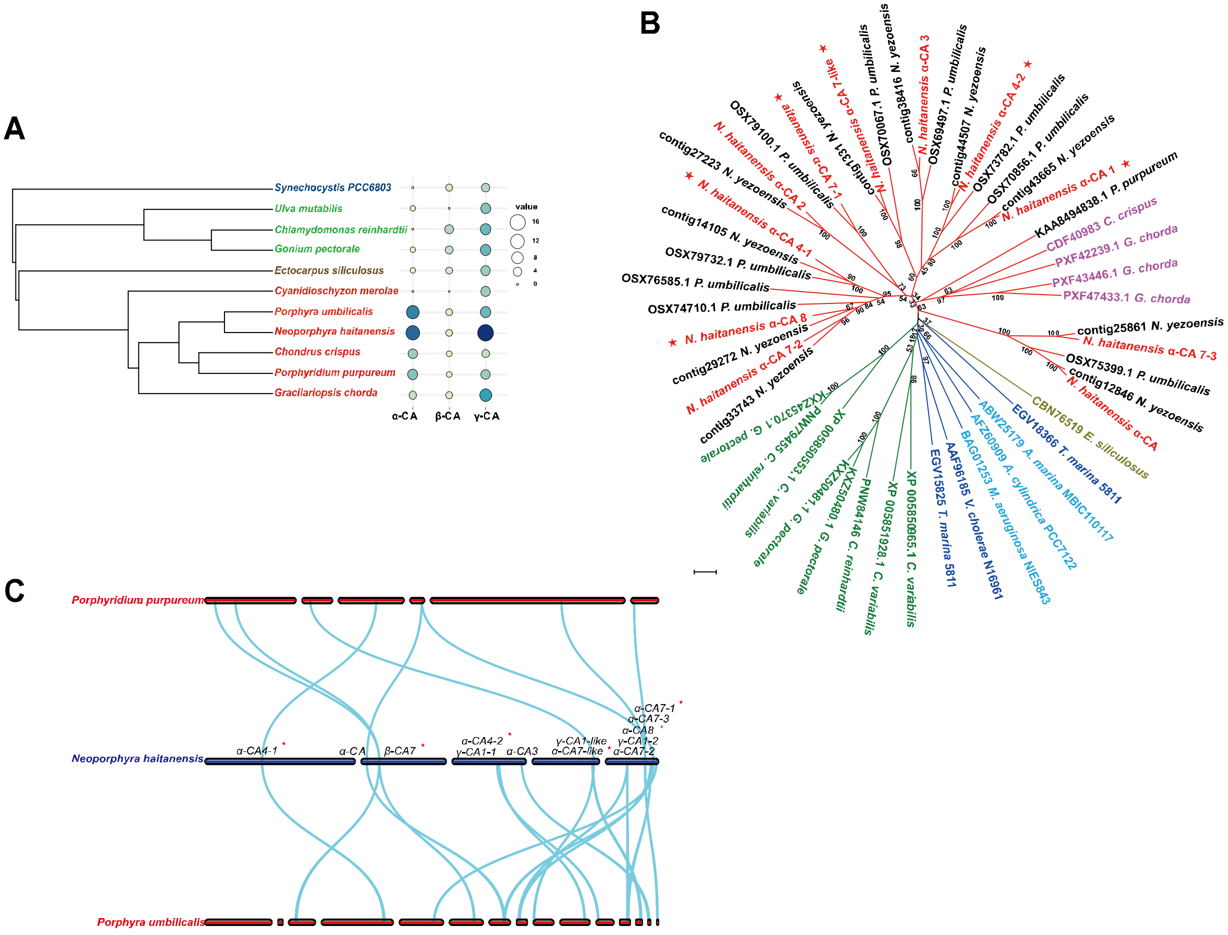

Supplementary fig. 12. Comparative analysis of carbonic anhydrase (CA) genes. (A) Phylogenetic analysis of CA genes in model photosynthetic algae. The copy numbers of genes for each CA subfamily are represented by the size of each circle. **(B)** Phylogeny of the α-CA genes and associated homologs. Neighbor-joining phylogenetic analysis of α-CA family members from red algae (red branches), brown algae *Ectocarpus siliculosus* (brown branches), green algae (green branches), and bacteria (blue branches) was conducted in MEGA7 with 1,000 bootstraps used to evaluate node support. All homologs from the *Neoporphyra haitanensis* genome are highlighted in red text. **(C)** Synteny analysis of CA genes between *N. haitanensis* vs. *Porphyra umbilicalis* in addition to *N. haitanensis* vs. *Neopyropia yezoensis*. Blue lines highlight the syntenic CA gene pairs between two species. Genes suspected of being derived from horizontal gene transfer (HGT) are marked with red stars.

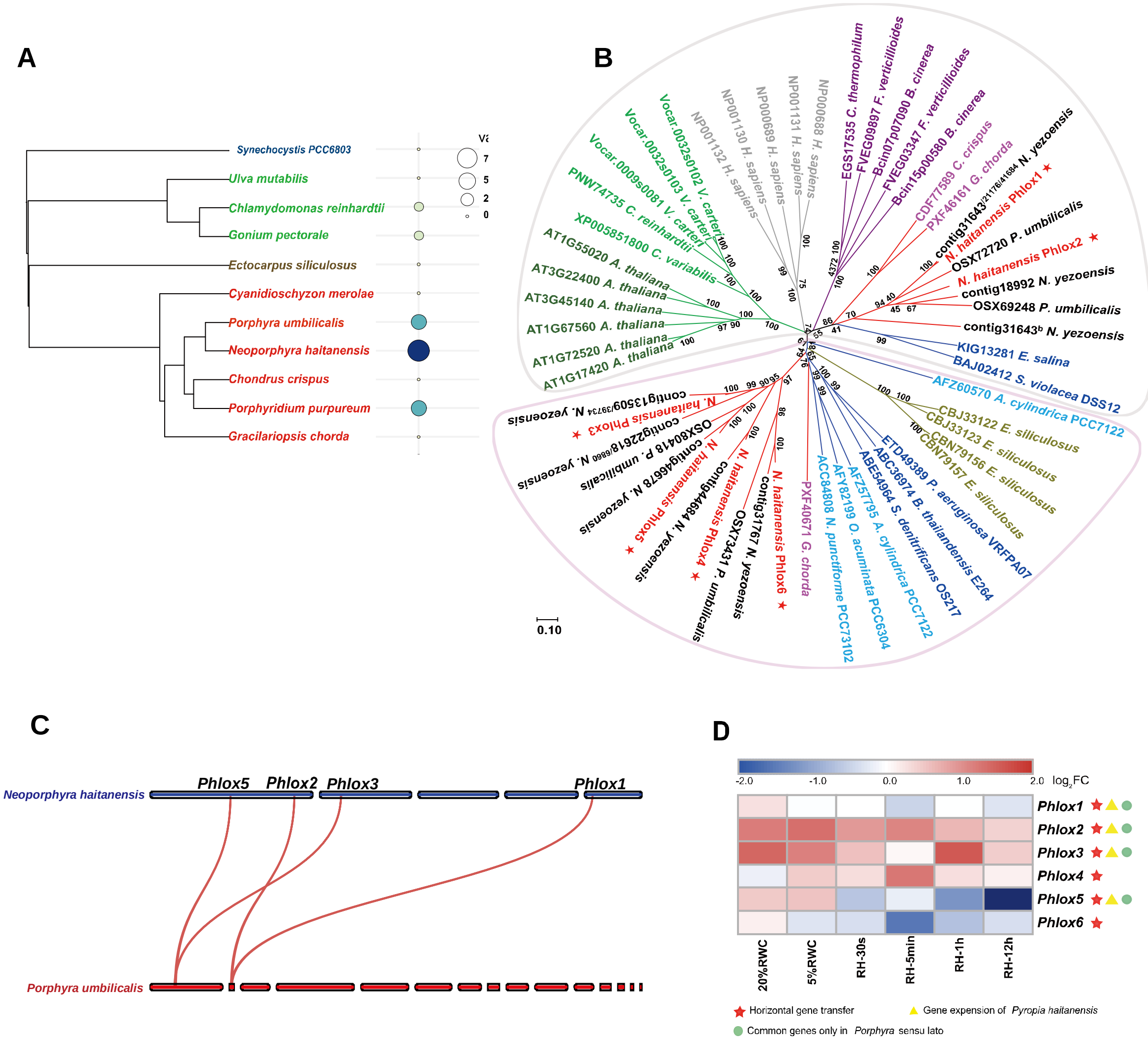

Supplementary fig. 13. Comparative analysis of lipoxygenase (Lox) genes in *Neoporphyra haitanensis*. (A) Phylogenetic analysis and copy numbers of Lox genes in algal genomes. The dendrogram was generated based on the phylogenetic tree constructed from single-copy orthologous gene sets identified in the *N. haitanensis* and other published algal genomes. The size of the circles indicates gene copy numbers. **(B)** Phylogenetic analysis of Lox gene families. Phylogenetic reconstructions were conducted using neighbor-joining methods in MEGA7 with 1,000 bootstrap replicates to evaluate node support. Red branches indicate red algal genes, green branches indicate plant genes (including those from green algae and *Arabidopsis thaliana*), blue branches indicate bacterial genes, brown branches indicate genes from *Ectocarpus siliculosus*, purple branches indicate fungal genes, and gray branches indicate genes from *Homo sapiens*. *N. haitanensis* homologs are highlighted in red text, and members suspected of deriving from HGT are indicated with red stars **(C)** Synteny analysis of Lox genes between *N. haitanensis* and *Porphyra umbilicalis* genomes. Red lines highlight syntenic Lox gene pairs between the two species. **(D)** Transcriptional profiles of Lox genes in *N. haitanensis* in response to osmotic stress. Heatmap shows the $\log_2$ fold-change values of transcripts. Fold change was calculated as FPKM (x% RWC or rehydration (RH) / FPKM (hydration control).

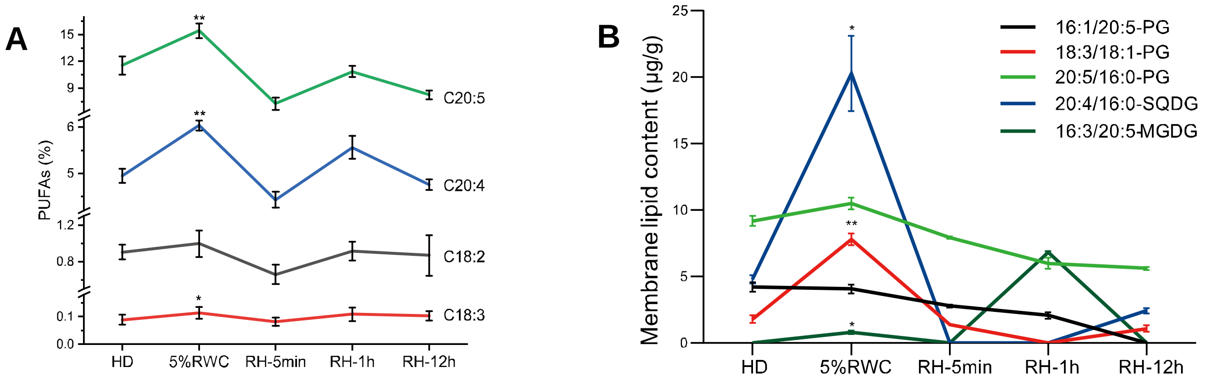

Supplementary fig. 14. Changes in lipid levels across dehydration and rehydration treatments. The panels show polyunsaturated fatty acid (PUFA) contents **(A)** and the contents of membrane lipids with PUFA **(B)** in *Neoporphyra haitanensis* under dehydration and rehydration conditions. Data show the means ± SD of three biological replicates. (**: p<0.01).

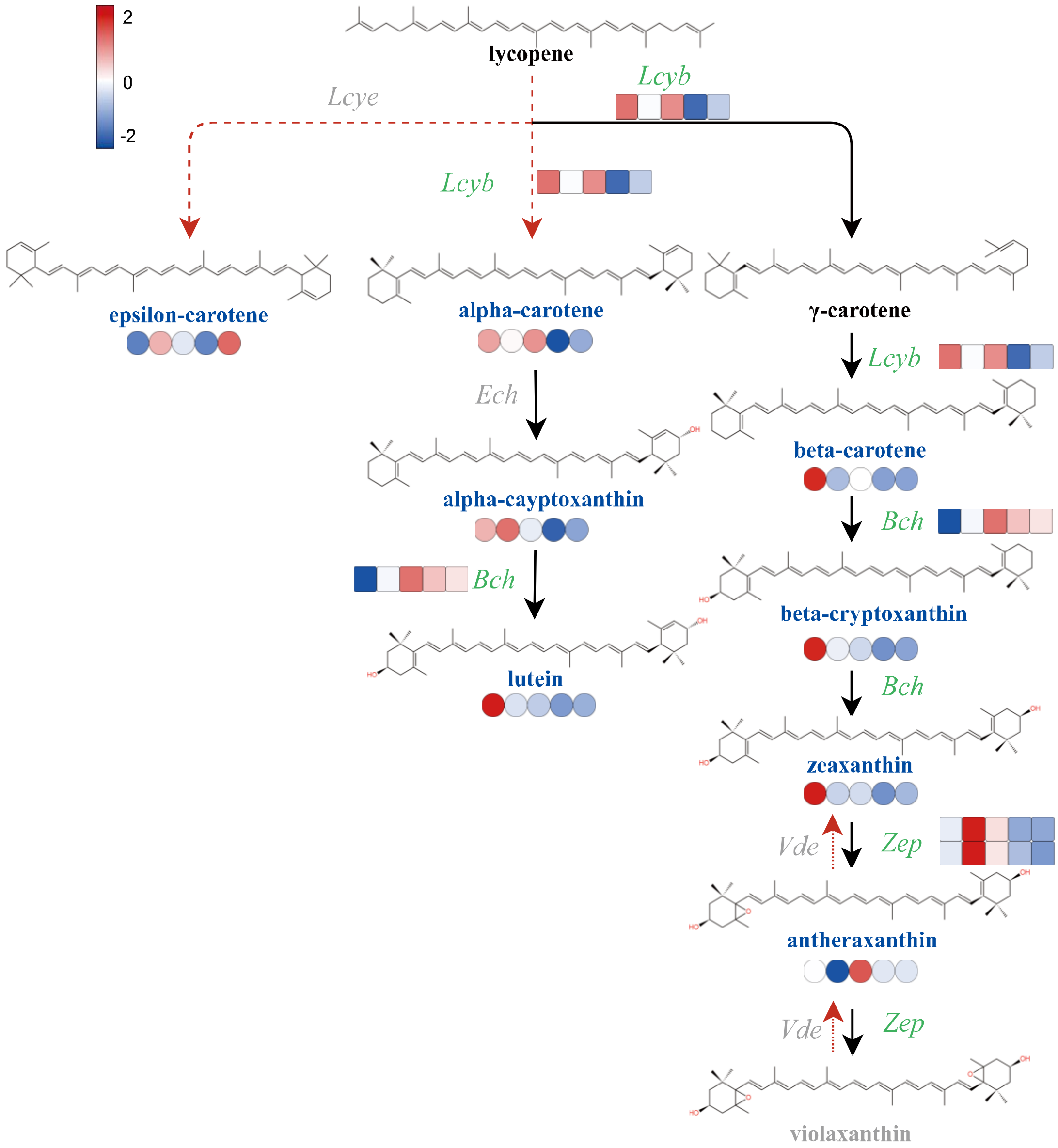

Supplementary fig. 15. Schematic representation of carotenoid biosynthesis in *Neoporphyra haitanensis*. Square heatmaps show the expression profiles of catalytic enzyme encoding genes for each reaction. The average FPKM values of each gene (n = 3) for different groups are shown in the heatmaps following z-score normalizations. Circular heatmaps represent the normalized average peak areas for carotenoids. Scaled expression profiles show the expression for each dehydration and rehydration sample collected (from left to right: HD (hydration), 5% RWC, RH (rehydration)-5min, RH-1h, RH-12h). Metabolites or genes indicated in gray were not detected.

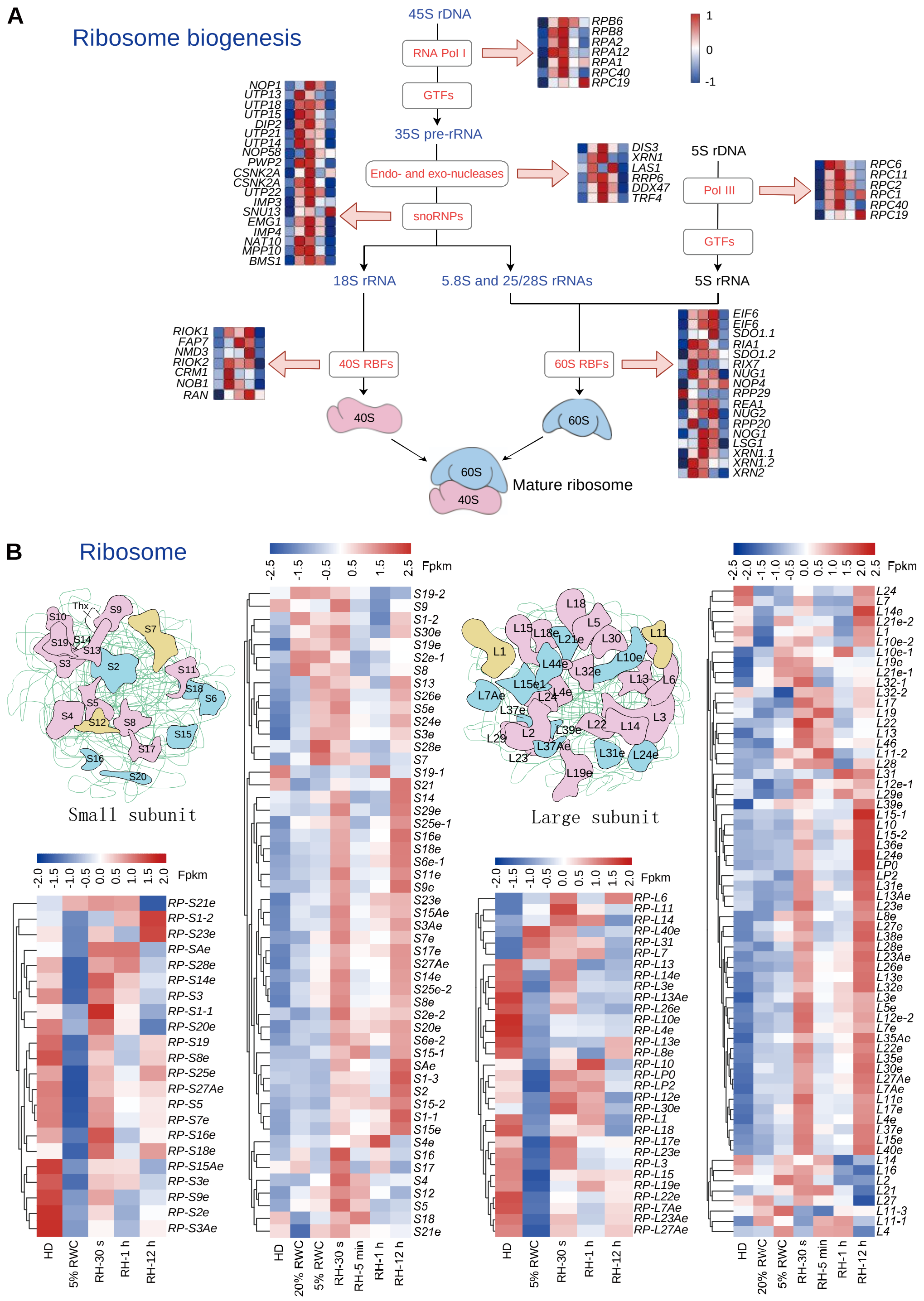

Supplementary fig. 16. Expression patterns of ribosomal biogenesis genes and ribosomal genes in *Neoporphyra haitanensis* during desiccation and rehydration cycling. (A) Schematic of ribosomal biogenesis activity differences across treatments. Transcriptome profiles are shown from the hydration (HD), 5% RWC, RH (rehydration)-30s, RH-1h, and RH-12h groups (from left to right). **(B)** Schematic of ribosome activity differences across treatments. FPKM values of genes in different groups were used to construct the heatmaps after z-score normalization (n = 3), while the normalized peak areas of proteins (n = 8) were used for the protein heatmaps.

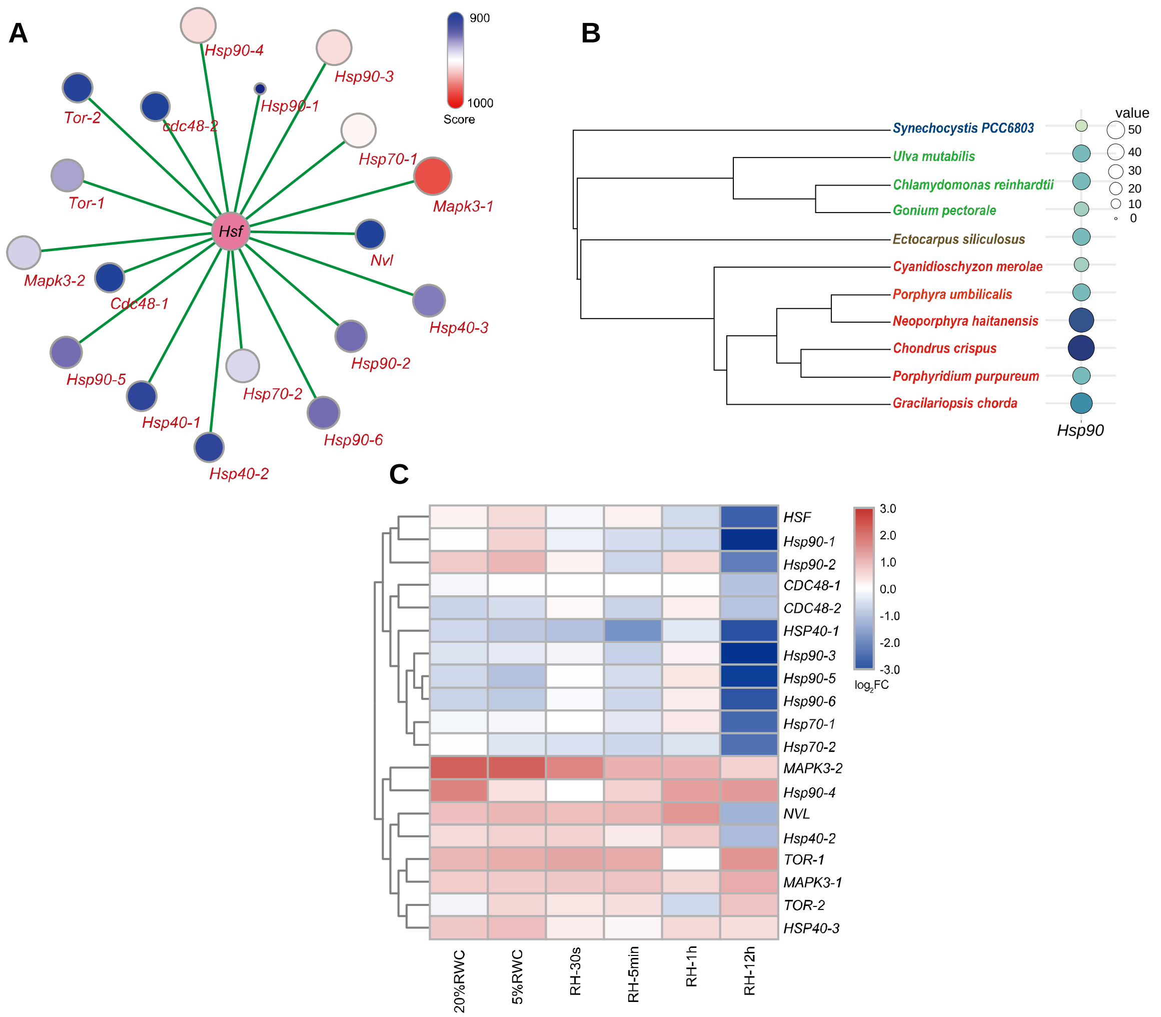

Supplementary fig. 17. Comparison of regulatory proteins that interact with Hsf in *Neoporphyra haitanensis* and their changes under osmatic stress. (A) Network analysis of Hsf and associated interacting proteins, as visualized with the Cytoscape software package. The sizes and colors of the nodes indicate the strength of the association. **(B)** Copy numbers of Hsp90 genes in algal genomes. The dendrogram was generated based on the phylogenetic reconstruction of single-copy orthologous genes identified in *N. haitanensis* and the other published algal genomes. Circle sizes represent gene copy numbers. **(C)** Heatmap showing the variation in expression profiles of the Hsf-interacting protein genes during desiccation and rehydration conditions. The dendrogram was constructed from hierarchical clustering of expression values. Fold change was calculated as FPKM (x% RWC or rehydration (RH) / FPKM (hydration control).

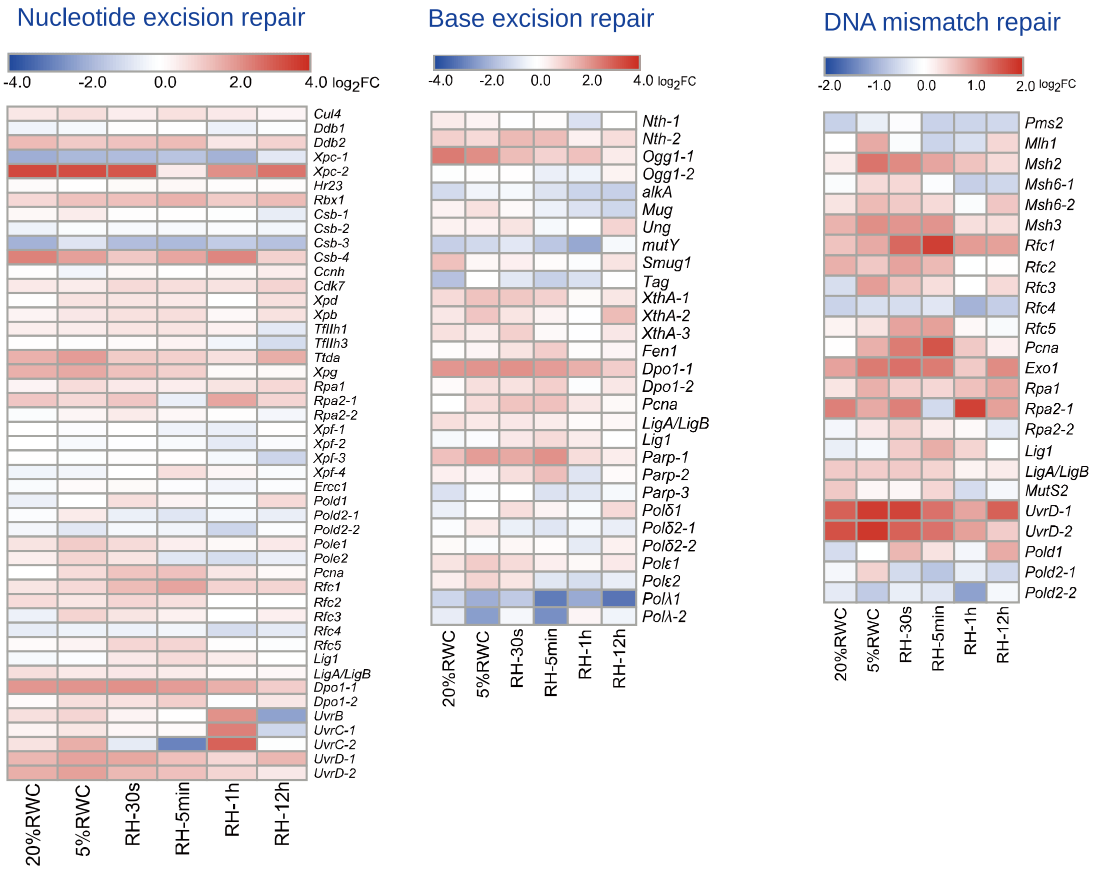

Supplementary fig. 18. Variation in differentially expressed genes related to nucleic acid repair in *Neoporphyra haitanensis*. Heatmap shows the log_2 fold-change values of transcripts. Fold change was calculated as FPKM (x% RWC or rehydration (RH) / FPKM (hydration control).

Supplementary table 1. Comparative genome analysis summary of whole assembly and *Neoporphyra haitanensis* genome assembly data.

Type	Whole assembly	*N. haitanensis* genome
Assembly size (bp)	99,100,156	49,666,066
Number of sequences	15 scaffolds + 343 contigs	5 scaffolds + 141 contigs
Contig N50 (bp)	965,843	650,156
Scaffold N50 (bp)	6,736,187	7,796,351
Longest sequence length (bp)	7,339,730	1,706,463
BUSCO (C+F) [a]	79.9%	80.2%

[a] Note: C: complete; F: fragment

Supplementary table 2. Repetitive element annotation and statistics for the *Neoporphyra haitanensis* genome.

Element annotation method	Repeat size (bp)	% of genome
Trf	6,980,473	14.05
Repeatmasker	2,358,082	4.75
Proteinmask	876,054	1.76
De novo	11,818,041	23.8
Total	15,699,264	31.61

Supplementary table 3. Summary of transposable element (TE) prevalence and other repetitive sequences in the *Neoporphyra haitanensis* genome.

Type	RepeatMasker TEs		RepeatProteinMask TEs		*De novo*		Combined TEs	
	Length (bp)	% of Genome	Length (bp)	% of Genome	Length (bp)	% of Genome	Length (bp)	% of Genome
DNA	298,145	0.6	18,390	0.04	649,940	1.31	924,847	1.86
LINE	108,473	0.22	4,569	0.01	226,806	0.46	335,683	0.68
SINE	821	0	0	0	127,320	0.26	128,031	0.26
LTR	719,412	1.45	853,095	1.72	4,817,098	9.7	5,118,009	10.3
Other	0	0	0	0	0	0	0	0
Unknown	2,529	0.01	0	0	5,300,816	10.67	5,303,290	10.68
Total TE	951,674	1.92	876,054	1.76	10,892,552	21.93	11,050,151	22.25

Supplementary table 4. Summary statistics for the *Neoporphyra haitanensis* genome assembly.

Sequence id	Sequence length (bp)	Gene number	Gene number per Kbp	Average gene length (bp)	Average CDS length (bp)
Superscaffold1	15,251,163	3,229	0.21	2,482.13	1,356.96
Superscaffold2	8,320,192	1,823	0.22	2,731.21	1,497.55
Superscaffold3	7,796,351	1,588	0.20	2,760.28	1,508.30
Superscaffold4	6,736,187	1,442	0.21	2,644.75	1,476.80
Superscaffold5	5,410,679	1,133	0.21	2,671.30	1,495.54

Sequence id	Average exon number per gene	Average exon length (bp)	Average intron length (bp)	Repeat size (bp)	Repeat% for Superscaffold
Superscaffold1	2.05	947.70	517.30	5,183,861	33.99
Superscaffold2	2.12	1,066.07	421.22	1,868,298	22.45
Superscaffold3	2.03	1,127.40	451.78	2,103,919	26.99
Superscaffold4	2.09	1,021.34	464.98	1,527,883	22.68
Superscaffold5	2.13	1,026.28	427.55	1,341,680	24.80

Note: 281 genes were identified on non-plastid unanchored contigs.

Supplementary table 5. Identification of BUSCO gene sets in *N. haitanensis* gene annotation.

**Type**	**Number**	**% Completeness**
Complete hit BUSCOs	233	76.9%
Complete single BUSCOs	226	74.6%
Complete duplicated BUSCOs	7	2.3%
Fragmented BUSCOs	27	8.9%
Missing BUSCOs	43	14.2%
Total	303	100%

Supplementary table 6. Comparative genomic assembly of several *Porphyra* species.

**Type**	**Genome size (Mbp)**	**Contig N50 (Kb)**	**Scaffold N50 (Mb)**	**Genes**
Neoporphyra haitanensis	49.66	650	7.8	9,496
Pyropia haitanensis PH40	53.3	510.3	5.8	10,903
Porphyra umbilicalis	87.7	137	130	12,287

Supplementary table 7. Alignment information for bacterial sequences isolated from the *Neoporphyra haitanensis* genome assembly.

Sequence Id	Sequence length	BLAST length	BLAST length ratio	Unaligned ratio
Superscaffold1	4,440,738	2,754,666	62.03%	37.97%
Superscaffold2	4,185,009	2,385,673	57.01%	42.99%
Superscaffold3	7,816,284	163,417	2.09%	97.91%
Superscaffold4	4,478,122	4,060,140	90.67%	9.33%
Superscaffold5	7,339,730	9,954	0.14%	99.86%
Superscaffold6	3,119,063	765,832	24.55%	75.45%
Superscaffold7	1,391,435	53,975	3.88%	96.12%
Superscaffold8	3,839,700	598,745	15.59%	84.41%
Superscaffold9	3,734,931	3,081,891	82.52%	17.48%
Superscaffold10	3,738,885	45,530	1.22%	98.78%
All	44,083,897	13,919,823	31.58%	68.42%

Supplementary Dataset (separate file).

Supplementary data 1. Assignment of contigs based on BlastN analysis.

Supplementary data 2. The common 142 gene families of *Neoporphyra haitanensis* and *Porphyra umbilicalis*.

Supplementary data 3. Typical horizontally transferred genes, *Porphyra* expended genes, and shared genes of *Neoporphyra haitanensis* and *Porphyra umbilicalis*.

Supplementary data 4. CheckM analysis of 10 bacterial superscaffolds.

Supplementary data 5. GTDB-Tk analysis of 10 bacterial superscaffolds.

Supplementary data 6. PATRIC annotation of 10 bacterial superscaffolds.

Supplementary data 7. Annotated phytohormone and vitamin synthesis-related genes in the 10 bacterial superscaffolds.

Supplementary data 8. Annotated genes shared among the bacterial sequences from the *Neoporphyra haitanensis* and other two *Porphyra* genomes.

Supplementary data 9. Candidate horizontal transferred genes in *Neoporphyra haitanensis*.

Supplementary data 10. The statistical data of gene family enrichment CAFÉ tests.

Supplementary References

Bligh EG, Dyer WJ. 1959. A rapid method of total lipid extraction and purification. *Can J Biochem Physiol*. 37:911–917.

Buchfink B, Xie C, Huson DH. 2014. Fast and sensitive protein alignment using DIAMOND. *Nat Methods* 12:59–60.

Camacho C, Coulouris G, Avagyan V, Ma N, Papadopoulos J, Bealer K, Madden TL. 2009. BLAST+: Architecture and applications. *BMC Bioinform*. 10:1-9.

Chaumeil PA, Mussig AJ, Hugenholtz P, Parks DH. 2020. GTDB-Tk: a toolkit to classify genomes with the Genome Taxonomy Database. *Bioinformatics* 36(6):1925-1927.

Davis JJ, Wattam AR, Aziz RK, Brettin T, Butler R, Butler RM, Chlenski P, Conrad N, Dickerman A, Dietrich EM, et al. 2020. The PATRIC bioinformatics resource center: expanding data and analysis capabilities. *Nucleic Acids Res.* 48(D1):D606-D612.

Edgar RC. 2004. MUSCLE: a multiple sequence alignment method with reduced time and space complexity. *BMC Bioinform.* 5:1-19.

Fan X, Qiu H, Han WT, Wang YT, Xu D, Zhang XW, Bhattacharya D, Ye NH. 2020. Phytoplankton pangenome reveals extensive prokaryotic horizontal gene transfer of diverse functions. *Sci Adv*. 6:eaba0111.

Gladyshev EA, Meselson M, Arkhipova IR. 2008. Massive horizontal gene transfer in bdelloid rotifers. *Science* 320:1210-1213.

Holt C, Yandell M. 2011. MAKER2: An annotation pipeline and genome-database management tool for second-generation genome projects. *BMC Bioinform*. 12:491.

Jorrin-Novo JV. 2014. Plant proteomics methods and protocols. *Methods Mol Biol*. 1072:3-13.

Jurka J. 2000. Repbase Update: A database and an electronic journal of repetitive elements. *Trends Genet*. 16:418–420.

Price MN, Dehal PS, Arkin AP. 2010. FastTree 2–approximately maximum-likelihood trees for large alignments. *PloS one* 5(3):e9490.

Qiu H, Price DC, Weber APM, Reeb V, Yang EC, Lee JM, Kim SY, Yoon HS, Bhattacharya D. 2013. Adaptation through horizontal gene transfer in the cryptoendolithic red alga *Galdieria phlegrea*. *Curr Biol*. 23(19): R865-R866.

Shaffer M, Borton MA, McGivern BB, Zayed AA, Rosa SLL, Solden LM, Liu PF, Narrowe AB, Rodríguez-Ramos J, Bolduc B, et al. 2020. DRAM for distilling microbial metabolism to automate the curation of microbiome function. *Nucleic Acids Res.* 48(16):8883-8900.

Slater GSC, Birney E. 2005. Automated generation of heuristics for biological sequence comparison. *BMC Bioinform*. 6:1-11.

Stanke M, Keller O, Gunduz I, Hayes A, Waack S, Morgenstern B. 2006. AUGUSTUS: ab initio prediction of alternative transcripts. *Nucleic Acids Res*. 34:W435–W439.

Tarailo-Graovac M, Chen NS. 2004. Using repeat masker to identify repetitive elements in genomic sequences. *Curr Protoc Bioinform*. 5:4-10.

Trapnell C, Pachter L, Salzberg SL. 2009. TopHat: Discovering splice junctions with RNA-Seq. *Bioinformatics* 25:1105–1111.

Trapnell C, Williams BA, Pertea G, Mortazavi A, Kwan G, van Baren MJ, Salzberg SL, Wold BJ, Pachter L. 2010. Transcript assembly and quantification by RNA-Seq reveals unannotated transcripts and isoform switching during cell differentiation. *Nat Biotechnol*. 28:511–515.

Wang DM, Yu XZ, Xu KP, Bi GQ, Cao M, Zelzion E, Fu CX, Sun PP, Liu Y, Kong FN, et al. 2020. *Pyropia yezoensis* genome reveals diverse mechanisms of carbon acquisition in the intertidal environment. *Nat Commun*. 11(1):4028. Doi: 10.1038/s41467-020-17689-1.

Wisniewski JR. Zougman A. Nagaraj N. Mann M. 2009. Universal sample preparation method for proteome analysis. *Nat Methods* 6(5):359-360.

Wu TD, Watanabe CK. 2005. GMAP: A genomic mapping and alignment program for mRNA and EST sequences. *Bioinformatics* 21:1859–1875.
